# Controlled depolymerization of cellulose by light-driven lytic polysaccharide oxygenases

**DOI:** 10.1038/s41467-020-14744-9

**Published:** 2020-02-14

**Authors:** Bastien Bissaro, Eirik Kommedal, Åsmund K. Røhr, Vincent G. H. Eijsink

**Affiliations:** 1 Faculty of Chemistry, Biotechnology and Food Science, Norwegian University of Life Sciences (NMBU), 1432 Ås Oslo, Norway; 2 INRAE, Aix Marseille University, UMR1163 Biodiversité et Biotechnologie Fongiques, 13009 Marseille, France

**Keywords:** Biocatalysis, Enzyme mechanisms

## Abstract

Lytic polysaccharide (mono)oxygenases (LPMOs) perform oxidative cleavage of polysaccharides, and are key enzymes in biomass processing and the global carbon cycle. It has been shown that LPMO reactions may be driven by light, using photosynthetic pigments or photocatalysts, but the mechanism behind this highly attractive catalytic route remains unknown. Here, prompted by the discovery that LPMOs catalyze a peroxygenase reaction more efficiently than a monooxygenase reaction, we revisit these light-driven systems, using an LPMO from *Streptomyces coelicolor* (*Sc*AA10C) as model cellulolytic enzyme. By using coupled enzymatic assays, we show that H_2_O_2_ is produced and necessary for efficient light-driven activity of *Sc*AA10C. Importantly, this activity is achieved without addition of reducing agents and proportional to the light intensity. Overall, the results highlight the importance of controlling fluxes of reactive oxygen species in LPMO reactions and demonstrate the feasibility of light-driven, tunable enzymatic peroxygenation to degrade recalcitrant polysaccharides.

## Abstract (web summary)

Lytic polysaccharide (mono)oxygenases (LPMOs) perform oxidative cleavage of polysaccharides. Here, the authors showed that the light-driven activity of LPMOs is dependent on hydrogen peroxide availability and can be controlled via the light intensity provided.

## Introduction

Environmental threats and future shortage in fossil-based energy and chemicals call for the development of sustainable processes for converting renewable sources into carbon and energy. Plant biomass represents an abundant source of renewable material, mainly in the form of polysaccharides in plant cell walls. However, the co-polymeric and recalcitrant nature of these cell walls constitutes a major hurdle in the extraction and valorization of the carbohydrate building blocks. Chitin represents another abundant, but recalcitrant source of renewable material, found in e.g. the shells of insects and crustaceans. Facing the structural intricacies of these biomaterials, plant-degrading and chitin-degrading microorganisms have developed complex arsenals of chemical and enzymatic tools for their deconstruction. Among the enzymatic tools are enzymes today known as lytic polysaccharide monooxygenases (LPMOs), which play a major role in biomass conversion by oxidative cleavage and, thus, structural disruption of biopolymers such as chitin^1,2^, cellulose^3–5^, as well as co-polymeric structures made of cellulose and hemicelluloses^6–8^. This disruptive action boosts the depolymerizing action of glycoside hydrolases^1,2,5^. LPMOs, classified today in families 9–11 and 13–16 of the auxiliary activities (AA) in the Carbohydrate Active enZymes database^9^, are ubiquitous enzymes with key roles in biological conversion of biomass by fungi and bacteria, but also with suggested roles in microbial pathogenicity^10–12^. LPMOs contribute to the efficiency of modern commercial cellulase cocktails used at industrial scale^13,14^.

**Fig. 1:**
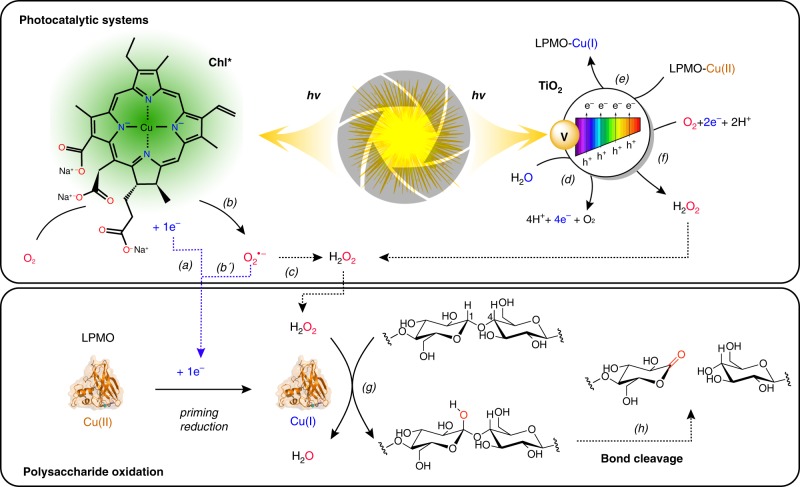
Light-driven LPMO-catalyzed oxidation of cellulose. For the Chl-based system, Cannella et al.^16^ proposed that photo-excited electrons activate the LPMO (*a*) and that reductants such as AscA or lignin could serve as electron donor to regenerate Chl (not shown). Here, we provide evidence for an alternative reduction pathway, where O_2_^•−^, produced via single electron reduction of O_2_ by photo-excited Chl (Chl*) (*b*), acts as reductant (*b’*). Disproportionation of O_2_^•−^ to H_2_O_2_ (*c*) can occur spontaneously or be accelerated by enzymatic action (e.g., superoxide dismutase, SOD) or by chemical reactions, such as reduction by AscA. In the V-TiO_2_ based system, reducing equivalents (in the form of excited electrons) are derived from V-TiO_2_-mediated and light-promoted oxidation of water (*d*). Several reduction reactions can occur at the surface of V-TiO_2_, including reduction of LPMO-Cu(II) to LPMO-Cu(I) (*e*^17^), reduction of O_2_ to H_2_O_2_ (*f* ^49,66^) or reduction of H_2_O_2_ to H_2_O (not shown^67^). The lower panel shows that the LPMO reduced by either of the two systems uses the generated H_2_O_2_ for cellulose oxidation (*g*). Hydroxylation of the glycosidic bond carbon leads to spontaneous bond cleavage (*h*^30,68^). Of note, these schemes focus on H_2_O_2_-driven LPMO catalysis, whereas O_2_-driven LPMO catalysis is not considered. See the main text for further details. The pigment shown in the top panel is the trisodium copper chlorin e_6_, the main component of chlorophyllin (*Chl*).

The use of light as a cheap energy source represents a key pillar of the emerging bioeconomy. Although the field of photocatalysis has been explored for decades, the field of photobiocatalysis, i.e., catalysis at the cross roads between photocatalysis and enzymology has been emerging more recently^15^. Harnessing the energy carried by visible light to drive biochemical processes, including enzymatic reactions, under eco-friendly conditions, constitutes a potentially valuable addition to currently available biotechnological tools. LPMO action requires energy in the form of reducing equivalents, and, in 2016, two studies were published that address the possibility of driving LPMO-catalyzed biomass conversion by light (Fig. 1)^16,17^. Cannella et al. showed that upon photo-excitation of pigments (e.g., chlorophyllin, *Chl*), and, notably, in the presence of a reductant, the activity of AA9E from *Thielavia terrestris* (*Tt*AA9E) on amorphous cellulose (PASC) could be boosted by up to two orders of magnitude^16^. In the same year, Bissaro et al. showed that a photocatalyst (vanadium-doped titanium dioxide, V-TiO_2_), catalyzing the thermodynamically challenging oxidation of water upon exposure to visible light, could generate the necessary reducing equivalents for oxidation of crystalline cellulose (Avicel) by AA10C from *Streptomyces coelicolor* (*Sc*AA10C)^17^. Both studies discussed possible mechanistic scenarios behind the observed effects, but the underlying mechanisms of both systems remain to be clarified.

Recently, there have been major developments in our understanding of the LPMO mechanism. LPMOs are mono-copper enzymes that catalyze the hydroxylation of the C1 and/or the C4 carbon in scissile glycosidic bonds^4,18^ (Fig. 1). Since the seminal study by Vaaje-Kolstad et al.^2^, LPMOs have been considered as monooxygenases, requiring the delivery of O_2_, two electrons and two protons for each catalytic cycle (R–H + O_2_ + 2e^−^ + 2 H^+^ → R–OH + H_2_O) via different putative reaction pathways^19,20^. In laboratory conditions, LPMO reactions are typically fueled by dissolved oxygen and added external reductants, such as ascorbic acid (AscA)^2^. Subsequently to the publication of the photobiocatalytic studies mentioned above^16,17^, we showed for one AA9-type and two AA10-type LPMOs that these enzymes, when supplied with both O_2_ and H_2_O_2_, preferentially use H_2_O_2_ as co-substrate. This observation led us to suggest that the previously described need for O_2_ may reflect the fact that O_2_ is a precursor of H_2_O_2_ in standard aerobic conditions typically used in LPMO reactions^21,22^. The reaction with H_2_O_2_ is best described as that of a peroxygenase (R–H + H_2_O_2_ → R–OH + H_2_O), where the oxygen atom, the two protons and the reducing equivalents are supplied simultaneously in the form of H_2_O_2_ to an activated LPMO (i.e., the reduced LPMO-Cu(I) state). Subsequently, several experimental studies have confirmed the efficiency of H_2_O_2_-driven LPMO reactions for different LPMO/substrate systems^23–29^. Furthermore, modeling studies have demonstrated the feasibility of the peroxygenase reaction^23,30–33^.

Clearly, this recent paradigm change calls for a re-investigation of light-driven cellulose oxidation by LPMOs, in particular the potential underlying role of reactive oxygen species (ROS). Here, we use coupled enzymatic assays to probe the roles of superoxide (O_2_^•−^) and H_2_O_2_ in the oxidation of Avicel by *Sc*AA10C fueled by visible light-exposed Chl or V-TiO_2_ (Fig. 1). We reveal the chemistry underlying light-driven LPMO action and show that light alone can drive LPMO catalysis. These results suggest avenues towards sustainable exploitation of mono-copper catalysts.

## Results

### Properties of chlorophyllin

Chlorophyllin (Chl), a water-soluble derivative of chlorophyll, is composed of a porphyrin ring metallated with copper (Fig. 1; see Methods section). We verified that the used commercial Chl has the expected absorbance and fluorescence properties, in part by comparison with copper-deficient chlorin e_6_ and Cu(II)-reconstituted chlorin e_6_ (Supplementary Fig. 1). The UV–Vis absorbance spectrum of Chl was nearly identical to that of Cu(II)-reconstituted chlorin e_6_, suggesting that the latter metallated species is the main constituent of the Chl powder (Supplementary Fig. 1a). Likewise, reconstituted Cu(II)-chlorin e_6_ and Chl showed equivalent fluorescence spectra (Supplementary Fig. 1b), with (expected) sharp excitation (*λ*^max^ = 343 nm) and emission peaks (*λ*^max^ = 685 nm), corroborating the fact that the photoactive species present in the Chl powder is Cu(II)-chlorin e_6_.

It has been shown that photo-excited chlorophyll has a very low reduction potential (*E*_0_ = −0.55 V vs. SHE^34^) and that saponified chlorophyll can catalyze the single electron reduction of O_2_ into O_2_^•−^^35^, which requires a strong reductant (*E*_0_ = −0.33 V vs. SHE^36^). However, no similar data exist for Chl, which displays a molecular structure similar to saponified chlorophyll but binds copper instead of magnesium.

To assess the redox properties of non photo-excited Chl species, we performed square-wave voltammetry experiments (Supplementary Table 1 and Supplementary Fig. 2) and found that the reduction potentials, *E*_p_, of Chl, chlorin e_6_ and metallated chlorin e_6_ were all in the range of 0.6–0.7 V vs. SHE, in accordance with the reduction potentials for Chl and non-metallated chlorin e_6_ reported by Novak and Komorsky-Lovric^37^. The measured reduction potentials of non photo-excited Chl species indicate that the LPMO in this study, *Sc*AA10C-Cu(II) (*E*_0_ ~ 0.236 ± 0.007 V vs. SHE^38^), cannot be reduced by these Chl species in absence of light.

### Fueling LPMO reactions with light-activated chlorophyllin

**Fig. 2:**
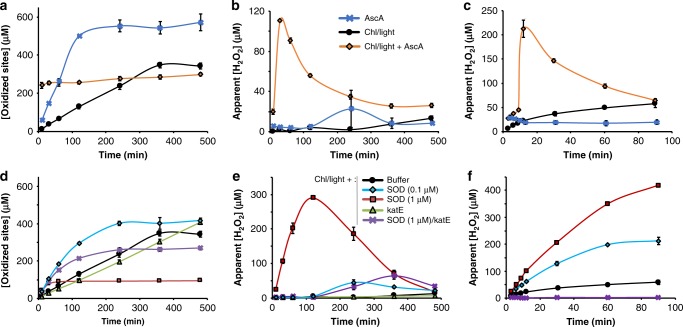
Probing the role of reactive oxygen species in the light/Chl/LPMO system. The graphs show time-courses for the release of aldonic acid products (**a**, **d**) and apparent H_2_O_2_ levels (**b**, **e**) in LPMO (*Sc*AA10C, 0.5 µM) reactions with substrate (Avicel, 10 g L^−1^), as well as apparent H_2_O_2_ levels in equivalent reactions lacking the LPMO (**c**, **f**). The top (**a**–**c**) panels show results for reactions fueled by Chl (500 µM) exposed to visible light (*I* = 25% *I*_max_, approx. 42 W cm^−2^) in the absence or presence of AscA (1 mM), or fueled by 1 mM AscA alone, in the dark. **d**–**f** Effect of SOD (0.1 or 1 µM) and catalase, katE (10 µg mL^−1^), on the reaction fueled by Chl/light. The legend code displayed in **b** applies also to **a**, **c**; likewise the legend code in **e** applies also to **d**, **f**. All reactions were carried out in sodium phosphate buffer (50 mM, pH 7.0) at 40 °C, under magnetic stirring. Before product quantification, solubilized cello-oligosaccharides were hydrolyzed by *Tf*Cel5A, to convert the LPMO products to a mixture of only two oxidized products with a degree of polymerization of 2 and 3 [GlcGlc1A, (Glc)_2_Glc1A], the amounts of which were summed up to yield the concentration of oxidized sites. It must be noted that the LPMO and the redox-active compounds in each of the reactions can engage in multiple side reactions, such as oxidation of AscA by H_2_O_2_ or generation of H_2_O_2_ by the LPMO, which explains why H_2_O_2_ levels are not stable and referred to as apparent. Error bars show ± s.d. (*n* = 3, independent experiments). Source data are provided as a Source Data file.

We first repeated experiments initially described by Cannella et al.^16^, i.e., combining AscA and Chl/light, using *Sc*AA10C and a lightning system we previously successfully used for the V-TiO_2_ system^17^. Indeed, use of the Chl/light-AscA system gave very high initial catalytic rates, notably accompanied by almost immediate enzyme inactivation (Fig. 2a; orange curve, see the 25% curve in Supplementary Fig. 3a, c for more details concerning the initial phase of the reaction). In accordance with the work by Cannella et al.^16^, the initial rates obtained with Chl/light-AscA were much higher than the rates obtained in a standard reaction with only AscA (Fig. 2a, blue curve). Yet, the standard reaction with only AscA produced more oxidized products compared to the reaction with Chl/light-AscA, because the LPMO stayed active for a much longer time (Fig. 2a). Interestingly, at the light intensities used here, which are considerably higher than those used by Cannella et al., the Chl/light system could also fuel the LPMO reaction in the absence of AscA, yielding relatively stable progress curves spanning several hours (Fig. 2a; black curve). Most importantly, this result shows that LPMO catalysis can take place with visible light as the only energy source (i.e., in the absence of added reducing power). Control experiments, including experiments in which the enzyme was replaced by various concentrations of Cu(II)SO_4_ (0–1000 µM), did not yield LPMO products (Supplementary Fig. 4).

Figure 2c shows that the Chl/light-AscA system alone (i.e., in absence of an LPMO) rapidly produces large amounts of H_2_O_2_ and Fig. 2b shows that H_2_O_2_ accumulates early in the LPMO reaction, at a time point where the LPMO is no longer active (as shown by the progress curve in Fig. 2a). Without added AscA, the system produces much lower amounts of H_2_O_2_, at a more regular pace (Fig. 2c), and H_2_O_2_ does not accumulate in the LPMO reactions (Fig. 2b), likely because it is consumed by the LPMO, which stays active over a long-time period (Fig. 2a). The reaction with only AscA provides a less clear picture, but it is clear that H_2_O_2_ is produced under these conditions (Fig. 2c), and it is worthwhile noting the small peak in the apparent level of accumulated H_2_O_2_ at 240 min in the LPMO reaction (Fig. 2b), i.e., just after the LPMO has become inactive (Fig. 2a). All in all, the data shown in Fig. 2a–c are compatible with the notion that LPMO activity is correlated with the availability of H_2_O_2_ and, that too high levels of H_2_O_2_ in LPMO reactions lead to enzyme inactivation and subsequent H_2_O_2_ accumulation. Control experiments in which fresh reaction components (Avicel, AscA and/or LPMO) were added to a reaction with AscA+Chl/light after 60 min of incubation (i.e., long after product formation had ceased, Fig. 2a) showed that only addition of fresh LPMO led to the reinitiation of product formation, confirming the impact of enzyme inactivation (Supplementary Fig. 5).

We then assessed whether the ability of the Chl/light system to drive LPMO reactions could be linked to the expected production of O_2_^•−^ by this system^35^. Superoxide dismutase (SOD) enzymatically converts O_2_^•−^ to H_2_O_2_ (Supplementary Fig. 6) and screening of a range of SOD concentrations showed that low amounts of SOD (100 nM) increased the LPMO initial rate and that higher amounts of SOD (up to 1 μM) led to almost immediate inactivation of the LPMO (Fig. 2d; more data in Supplementary Fig. 7). Accordingly, we observed that addition of 100 nM SOD led to increased H_2_O_2_ production (Fig. 2f) while yielding relatively low H_2_O_2_ accumulation in the complete system (Fig. 2e). On the other hand, addition of 1 μM SOD led to higher H_2_O_2_ levels (Fig. 2f) and accumulation of high levels of H_2_O_2_ in the reaction with the (rapidly inactivated) LPMO and substrate (Fig. 2e). These results indicate that the Chl/light system produces large amounts of O_2_^•−^ and that the degree of conversion O_2_^•−^ to H_2_O_2_, e.g., by SOD (Fig. 2d–f), determines both the catalytic rate and the stability of the LPMO.

Addition of catalase (katE), a H_2_O_2_ consuming enzyme (Supplementary Fig. 6), to the reaction containing a (too) high amount of SOD (1 μM) had a clear beneficial effect, leading to higher LPMO activity over a longer period (Fig. 2d) and little accumulation of H_2_O_2_ (Fig. 2f). Addition of catalase to the Chl/light system gave a stable LPMO reaction, with only a slight reduction in rate, no visible enzyme inactivation within the 12-h measuring period (Fig. 2d), and almost no accumulation of H_2_O_2_ (Fig. 2e). In light of the idea that H_2_O_2_ drives LPMO action, it may seem surprising that, while catalase expectedly abolished accumulation of H_2_O_2_ (Fig. 2f), it hardly affected, or even had a seemingly positive effect on, LPMO activity. There are, however, straightforward explanations for this paradox. Firstly, the beneficial effect of catalase on the reaction with 1 μM SOD is due to catalase removing the surplus of H_2_O_2_ that otherwise would lead to LPMO inactivation. Secondly, kinetic data^25,39^ show that a reduced LPMO in the presence of substrate will easily compete with catalase for available H_2_O_2_; it is thus plausible that, while catalase consumes produced H_2_O_2_ in the absence of the LPMO (Fig. 2f), H_2_O_2_ will primarily be consumed by the LPMO in reactions containing both enzymes and an LPMO substrate (Fig. 2d).

If the ability of the Chl/light system to drive LPMO reactions indeed is due to the production of O_2_^•−^, which is subsequently converted to H_2_O_2_, the huge effect of adding AscA (Fig. 2a–c) suggests that AscA catalyzes the otherwise spontaneous conversion of O_2_^•−^ to H_2_O_2_, as has indeed been shown (see ref. ^40^; Supplementary Fig. 6). Accordingly, the initial rate of LPMO catalysis and the degree of enzyme inactivation could be modulated by varying the amount of AscA (Supplementary Fig. 8).

**Fig. 3:**
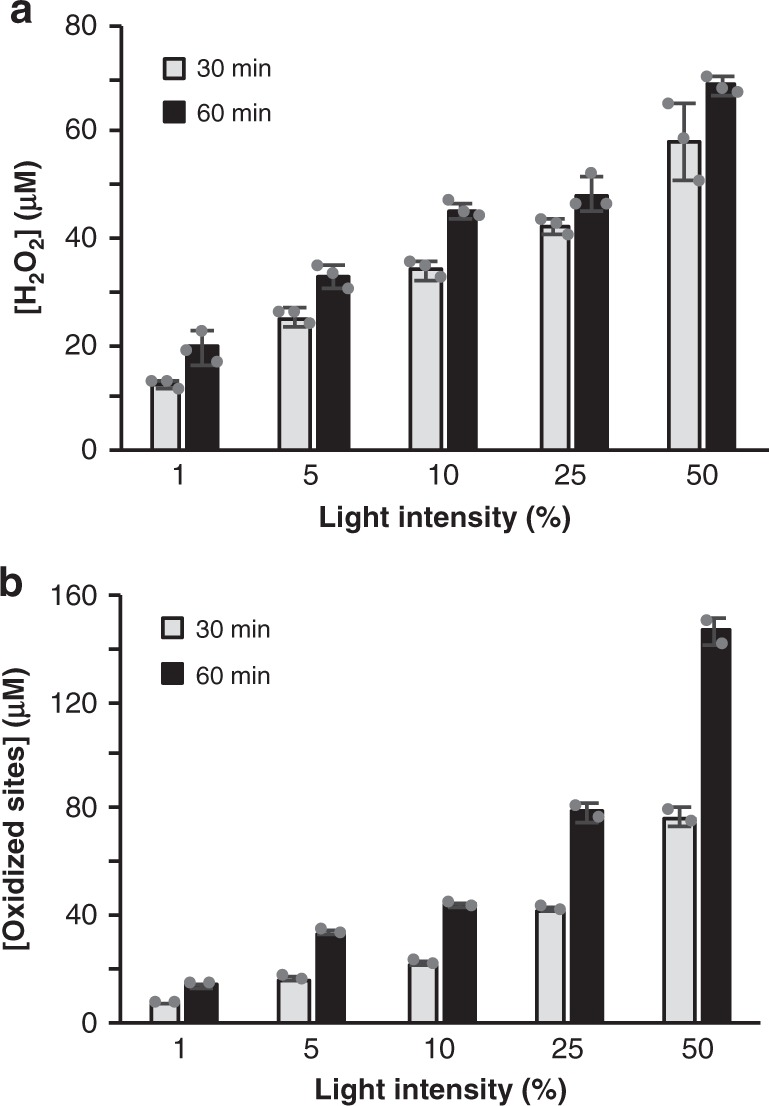
The effect of light intensity on H_2_O_2_ production and LPMO activity in the Chl-system. The graphs show, for different light intensities (100% = approx. 168 W cm^−2^), **a** the apparent quantity of H_2_O_2_ generated in reactions with Chl (500 µM)/light and Avicel and **b** the amount of soluble aldonic acid products released in identical reactions that also contained *Sc*AA10C (0.5 µM). See Supplementary Fig. 3b–d for complete time courses of *Sc*AA10C-catalyzed oxidation of Avicel. Note that direct quantitative comparison of the levels of produced H_2_O_2_ and oxidized products is not possible because only soluble LPMO products were quantified, and, more importantly, measured H_2_O_2_ levels reflect the net result of H_2_O_2_ production and H_2_O_2_–consuming side reactions, as discussed in the main text. All reactions were carried out in sodium phosphate buffer (50 mM, pH 7.0) at 40 °C, under magnetic stirring and contained 10 g L^−1^ Avicel. Before product quantification, solubilized cello-oligosaccharides were hydrolyzed by *Tf*Cel5A, to convert the LPMO products to a mixture of only two oxidized products with a degree of polymerization of 2 and 3 [GlcGlc1A, (Glc)_2_Glc1A], the amounts of which were summed up to yield the concentration of oxidized sites. Error bars show ± s.d. (*n* = 3 for **a** and *n* = 2 for **b**, independent experiments). Source data are provided as a Source Data file.

Likewise, there was a clear correlation between the light intensity, which determines the rate of O_2_^•−^ generation, and LPMO activity for both the Chl/light (Fig. 3 and Supplementary Fig. 3) and the Chl/light-AscA system (Supplementary Fig. 3). Figure 3 shows that the decrease in LPMO activity upon decreasing the light intensity applied to the Chl/light system (Fig. 3b) correlates with decreased production of H_2_O_2_ (measured in absence of the LPMO) (Fig. 3a). Importantly, in the absence of LPMO activity, H_2_O_2_ generated by Chl/light system will be further reduced, which implies that the measured apparent levels of H_2_O_2_ are likely an underestimation of the true levels of produced H_2_O_2_. On the other hand, when the LPMO and substrate are present, one could expect that the high affinity of the LPMO for H_2_O_2_ results in efficient integration of H_2_O_2_^25^ in oxidized reaction products. Accordingly, while Fig. 3a shows an apparent retardation in the rate of H_2_O_2_ production in the absence of the LPMO, Fig. 3b shows a linear increase in LPMO products over time.

Taken together, the data presented above are compatible with a scenario in which the ability of the Chl/light and Chl/light-AscA systems to drive LPMO reactions is due to the light-driven generation of O_2_^•−^, which is converted to H_2_O_2_. As shown by Fig. 2 and Supplementary Figs. 3, 7, 8 and discussed above, and in agreement with several studies published since the discovery of the role of H_2_O_2_ in LPMO catalysis^22,25–29,41^, H_2_O_2_ clearly is a double-edged sword: it can drive the LPMO reaction with high efficiency, but is also a potentially harmful entity if its levels are not controlled. Of note, while potentially harmful effects of H_2_O_2_ on enzymes due to non-specific oxidation reactions are well known, the deleterious effect of H_2_O_2_ on LPMOs is specific in the sense that the reaction of substrate-free, reduced LPMOs with H_2_O_2_ leads to oxidative damage in the catalytic center^22,42^.

The dualistic impact of H_2_O_2_ could also explain why our present conclusions differ from those of Möllers and colleagues^43^, who concluded that reactive oxygen species (ROS) are not part of light-driven LPMO catalysis. On the one hand, these authors observed that exposure of Chl/AscA to light leads to consumption of O_2_. In accordance with our present results, this effect was attributed to the conversion of O_2_ into O_2_^•−^ and H_2_O_2_, as shown by increases in O_2_ levels upon addition of SOD and catalase, respectively. On the other hand, Möllers et al. found that addition of catalase or SOD did not affect measured LPMO product levels, leading to the conclusion that the generated ROS do not affect LPMO catalysis. The absence of an effect of catalase on apparent LPMO activity is not surprising in light of kinetic considerations (see above). The absence of an effect of SOD could be due to the fact that Möllers et al. did not monitor LPMO activity over time and thus may have overlooked effects of LPMO inactivation. Perhaps, the authors encountered a situation similar to the one presented in Supplementary Fig. 9, where SOD effects are not visible because the LPMO becomes fully inactivated prior to the first sampling point. In our hands, SOD clearly has an effect on light-driven LPMO performance, as shown by Fig. 2d–f. Of course, one cannot exclude that *Tt*AA9E, the family AA9 LPMO used by Möllers et al., employs a different mechanism compared to *Sc*AA10C, in terms of both activation and inactivation.

### LPMO reduction by light-activated chlorophyllin

**Fig. 4:**
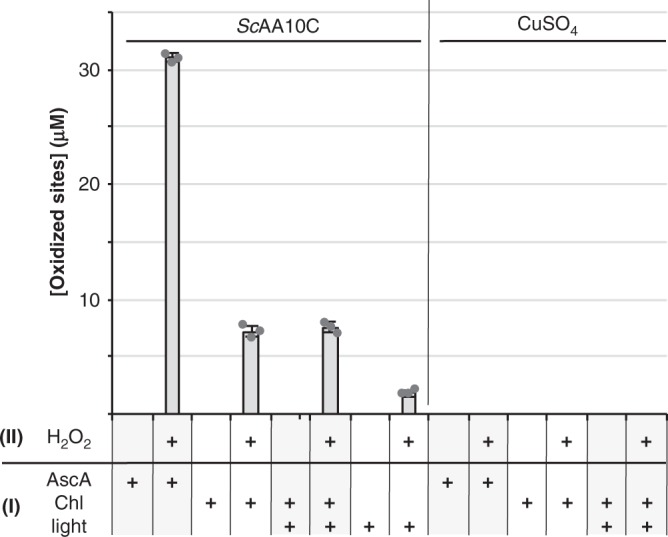
Anaerobic activation of *Sc*AA10C by Chl/Light. The experiment consisted of two phases, namely a pretreatment phase (I) followed by an activity test phase (II). Conditions were varied as indicated by the +signs below the graph, as further explained below. All phases were performed under anaerobic conditions, at 25 °C. During phase (I), *Sc*AA10C (50 µM; note the high concentration) or CuSO_4_ (50 µM; to assess the occurrence of transition metal-catalyzed non-enzymatic reactions) were incubated with either AscA (100 µM, i.e., a two-fold molar surplus relative to the LPMO) as positive control or Chl (500 µM) in sodium phosphate buffer (50 mM, pH 7.0), in sealed Quartz cuvettes, exposed or not to light (visible light, 25% *I*_max_, ca. 42 W cm^−2^). After 10 min of incubation (phase I), the pre-treated sample (50% of final volume) was mixed with a suspension of Avicel (10 g L^−1^ final) in sodium phosphate buffer (50 mM, pH 7.0) and, then, H_2_O_2_ (200 µM final) was added when indicated (“+” sign, otherwise replaced by water). Phase (II) reaction mixtures were incubated for 30 min before being heat-treated (100 °C, with shaking at 800 rpm) and filtered. Before product quantification, solubilized cello-oligosaccharides were hydrolyzed by *Tf*Cel5A, to convert the LPMO products to a mixture of only two oxidized products with a degree of polymerization of 2 and 3 [GlcGlc1A, (Glc)_2_Glc1A], the amounts of which were summed up to yield the concentration of oxidized sites Error bars show ± s.d. (*n* = 3, independent experiments). Source data are provided as a Source Data file.

Hydrogen peroxide-driven LPMO activity requires a priming reduction of the LPMO from the Cu(II) to the Cu(I) state^22,39^. The correlations between H_2_O_2_ availability and LPMO activity described above suggest that this priming reduction is not rate-limiting. This is supported by stopped-flow kinetics showing fast (4.2 × 10^5^ M^−1^ s^−1^) and full reduction of an AA10 LPMO when using as little as 5 µM AscA^23^. The situation may be different when using the Chl/light system, without added AscA. We have previously shown that superoxide, which is produced by the light-exposed Chl (Fig. 2), can serve as reductant^22^. Another option would be the direct reduction of the LPMO by photo-excited Chl, as suggested by Cannella et al.^16^. To probe this latter hypothesis, we initially attempted to monitor LPMO reduction by light-exposed Chl in anaerobic conditions using fluorescence^17^, but we did not manage to establish conditions that allowed informative fluorescence measurements. In an alternative experiment, carried out under anaerobic conditions we first exposed the LPMO to Chl/light and then added substrate and H_2_O_2_, while switching off the light. Figure 4 shows that this approach led to only very low LPMO activity, compared to a control reaction with AscA, and that this activity was independent of the application of light. Thus, it would seem that light-induced reduction of the LPMO does not occur in anaerobic conditions, which supports the idea that, under aerobic conditions, the LPMO is mainly reduced by (oxygen-derived) superoxide and not by direct electron transfer from Chl to the LPMO.

Of note, however, this issue remains ambiguous. Given the expected low reduction potential of photo-excited chlorophyllin, efficient reduction of the LPMO may occur. Thus, it is possible that in the two-phase experiment of Fig. 4, the LPMO gets reduced but is re-oxidized before being transferred to the substrate/H_2_O_2_ mixture. Indeed, measurement of reduction potentials of the ground state Chl (>0.6 V vs. SHE; see above), present in the first phase of the experiment, indicate that it can act as an oxidant of LPMO-Cu(I), which would regenerate LPMO-Cu(II). In a real (i.e., one-phase) experiment, LPMO-Cu(I) would have several possible fates: (i) re-oxidation in solution by either ground state Chl, by O_2_ or by H_2_O_2_, or, (ii) binding to the substrate and formation of a productive complex with the co-substrate, leading to substrate hydroxylation and cleavage. The distribution between these different pathways will depend on kinetics of the different reactions. Available kinetic studies predict that, in the presence of a suitable substrate, the productive substrate hydroxylation pathway will be favored^23,25^.

### Metalation of Chl affects H_2_O_2_ production and LPMO activity

**Fig. 5:**
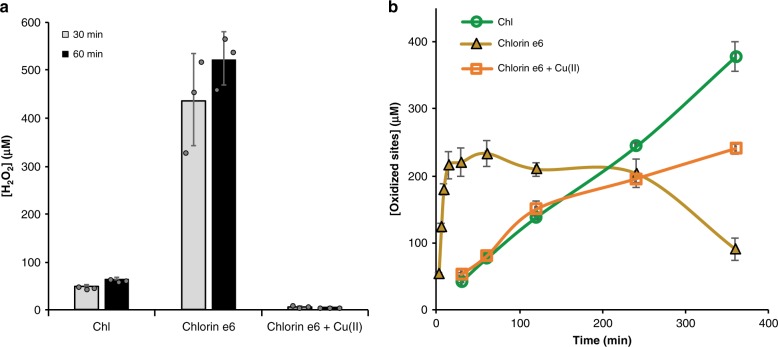
Effect of copper on H_2_O_2_ generation by light-exposed Chl and chlorin e_6_ and on light-driven LPMO activity. **a** H_2_O_2_ production by 500 µM of light-exposed Chl, chlorin e_6_ or chlorin e_6_ supplemented with 0.9 molar equivalents of Cu(II). **b** Time-course release of soluble aldonic acid products released from Avicel by *Sc*AA10C (1 µM) when fueled by light-exposed Chl, chlorin e_6_ or chlorin e_6_ complemented with 0.9 eq. Cu(II). All reactions were carried out with 500 µM pigment and exposed to visible light (*I* = 25% *I*_max_, approx. 42 W cm^−2^), in sodium phosphate buffer (50 mM, pH 7.0) at 40 °C, under magnetic stirring. Before product quantification, solubilized cello-oligosaccharides were hydrolyzed by *Tf*Cel5A, to convert the LPMO products to a mixture of only two oxidized products with a degree of polymerization of 2 and 3 [GlcGlc1A, (Glc)_2_Glc1A], the amounts of which were summed up to yield the concentration of oxidized sites. Error bars show ± s.d. (*n* = 3, independent experiments). Source data are provided as a Source Data file.

The UV–Vis and fluorescence analyses of chlorin e_6_ (Supplementary Fig. 1) showed that the photochemical properties of the porphyrin ring are modulated by copper-binding^44^. Measurements of H_2_O_2_ production rates by light-exposed chlorin e_6_ revealed a drastic effect of copper binding on apparent H_2_O_2_ production (Fig. 5a). Compared to light-exposed Chl, light-exposed Cu(II)-depleted chlorin e_6_ produced much more H_2_O_2_, whereas Cu(II)-reconstituted chlorin e_6_ produced much less H_2_O_2_. The fact that Cu(II) reconstituted-chlorin e_6_ yields less H_2_O_2_ than the commercial preparation of Chl suggest that the latter likely is not fully saturated with copper. Our results clearly show that metalation of the pigment is an important parameter to take into account for future studies. Testing of these three compounds in LPMO reactions showed product formation patterns akin to what we describe above, where Cu(II)-depleted chlorin e_6_ leads to very high LPMO activity and fast inactivation of the LPMO, while Chl and Cu(II)-chlorin e_6_ give lower LPMO reaction rates and less inactivation (Fig. 5b). As discussed above, direct comparison of LPMO activity and the apparent ability of the corresponding LPMO-deficient system to generate (and accumulate) H_2_O_2_ is complicated by the many possible side reactions. For instance, Fig. 5b shows that reactions containing Chl or Cu(II)-chlorin e_6_ yield equivalent LPMO activity (within the first 2 h) whereas the former system shows higher H_2_O_2_ accumulation in the absence of the LPMO (Fig. 5a). Still, Fig. 5 also points at a link between H_2_O_2_ generation and LPMO activity.

### The AscA-driven reaction is complex and uncontrolled

**Fig. 6:**
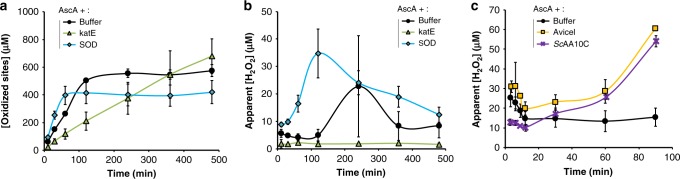
Characterization of AscA-driven cellulose oxidation by *Sc*AA10C. The graphs show time-courses for the release of aldonic acid products (**a**) and apparent H_2_O_2_ levels (**b**) upon incubating Avicel (10 g L^−1^) with *Sc*AA10C (0.5 µM) in presence of AscA (1 mM), in the dark. Reaction conditions varied in terms of the presence of SOD (0.1 µM) and katE (10 µg mL^−1^). The legend code is indicated in each panel. **c** Apparent H_2_O_2_ levels during incubation of AscA (1 mM) with either *Sc*AA10C (0.5 µM) or Avicel (10 g L^−1^) or with no addition (buffer). All reactions were carried out in sodium phosphate buffer (50 mM, pH 7.0) at 40 °C, under magnetic stirring. Before product quantification, solubilized cello-oligosaccharides were hydrolyzed by *Tf*Cel5A, to convert the LPMO products to a mixture of only two oxidized products with a degree of polymerization of 2 and 3 [GlcGlc1A, (Glc)_2_Glc1A], the amounts of which were summed up to yield the concentration of oxidized sites. Error bars show ± s.d. (*n* = 3, independent experiments). Source data are provided as a Source Data file.

For comparative purposes, and to further highlight the potential of the light-driven reactions described here, we also performed reactions with only AscA, a well-known and commonly used reductant to drive LPMO reactions^2,14,31^. The reactions yielded less clear-cut results (Fig. 6a–c) compared to the studies with Chl/light (Fig. 2), which is likely due to the many possible redox reactions involving AscA, superoxide and H_2_O_2_ (see Supplementary Fig. 6). However, the same overall trend stood out: both higher LPMO activity and faster apparent enzyme inactivation were correlated with higher H_2_O_2_ levels. As above, addition of a small amount of SOD gave a higher initial rate and faster inactivation. The addition of catalase yielded stable LPMO product formation over time (Fig. 6a), likely because catalase lowers the steady-state H_2_O_2_ concentration. By keeping apparent H_2_O_2_ levels low, the LPMO becomes less active, but is also less prone to inactivation.

On a side note, Fig. 6a illustrates the risk of assessing LPMO activity by measuring single time points. Assessment of the effect of catalase in the experiment shown in Fig. 6a at e.g., 120 min vs. e.g., 480 min would lead to opposite conclusions as to the effect of catalase on LPMO catalysis.

Figure 6c shows another complexity of reactions with added reductant, in that production of H_2_O_2_ occurred when mixing 1 mM AscA with only the substrate, Avicel (Fig. 6c). Similar amounts of H_2_O_2_ were measured when Avicel was replaced by *Sc*AA10C-Cu(II), where the latter is known to produce H_2_O_2_ when incubated with reductant and O_2_ in the absence of substrate^45^. These results indicate that in these AscA-driven reactions, the substrate contributes to the generation of H_2_O_2_, possibly because of the pro-oxidant properties of AscA that become apparent in the presence of free metals (see ref. ^46^ and below) that may be present in Avicel.

**Fig. 7:**
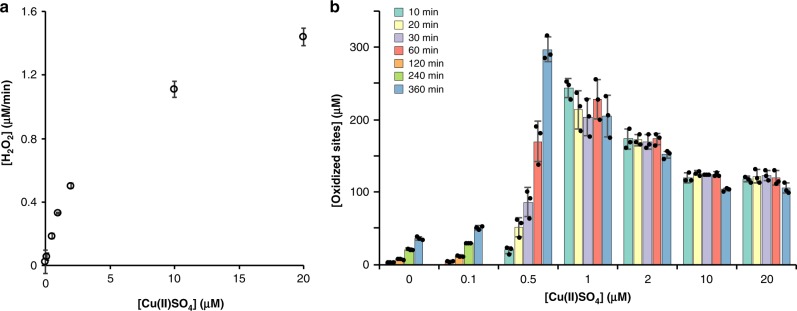
Effect of free copper on non-enzymatic H_2_O_2_ production and *Sc*AA10C activity. **a** The graph shows the rate of H_2_O_2_ production in reactions containing AscA (50 µM) and varying amounts of Cu(II)SO_4_ (0–20 µM), in sodium phosphate buffer (50 mM, pH 7.0), at 25 °C. **b** The graph shows the time course release of soluble oxidized products from Avicel (10 g L^−1^) by *Sc*AA10C (1 µM) in presence of AscA (1 mM) and varying amounts of Cu(II)SO_4_ (0–20 µM), in sodium phosphate buffer (50 mM, pH 6.0) and incubated in a thermomixer (1000 rpm, 40 °C). Note that soluble oxidized products were not detectable before 60 min incubation for reactions containing 0 µM and 0.1 μM of added free copper; therefore, the sampling was adapted (60, 120, 240, and 360 min). Before product quantification, solubilized cello-oligosaccharides were hydrolyzed by *Tf*Cel5A, to convert the LPMO products to a mixture of only two oxidized products with a degree of polymerization of 2 and 3 [GlcGlc1A, (Glc)_2_Glc1A], the amounts of which were summed up to yield the concentration of oxidized sites. Error bars show ± s.d. (*n* = 3, independent experiments). Source data are provided as a Source Data file.

Digging further into these complexities, we then tested the effect of the addition of free transition metals on the LPMO-independent production rate of H_2_O_2_ by AscA and on LPMO-catalyzed degradation of Avicel. Figure 7a shows that the rate of H_2_O_2_ production in AscA solutions increased drastically upon addition of CuSO_4_. Due to technical limitations of the assay, only 50 µM AscA was present in the reactions displayed in Fig. 7a; at commonly used higher AscA concentrations (mM range), H_2_O_2_ production rates will be much higher. Figure 7b shows that the effect of CuSO_4_ on H_2_O_2_ production rates is reflected in LPMO activity, with similar trends as those seen in e.g., the progress curves of Fig. 2: increased amounts of CuSO_4_ led to higher initial catalytic rates (e.g., 0.13 µM min^−1^ vs. at least 19 µM min^−1^ in reactions with no added CuSO_4_, vs. 1 µM CuSO_4_, respectively) and to faster inactivation. At the higher CuSO_4_ concentrations, the enzyme was already inactivated at the first measuring point. A contribution of free transition metals to H_2_O_2_ production in standard (i.e., O_2_- and reductant-driven) LPMO reactions is an important parameter to consider since these metals may be present in significant amounts that vary between substrates and enzyme preparations. Such variations will inevitably lead to variations in observed LPMO activities.

Overall, it is clear that steady LPMO reactions require strict control of the delivery of reducing equivalents and H_2_O_2_, and of redox side reactions. Such control is likely not achieved in typical AscA-driven and O_2_-driven reactions reported in the literature. On the other hand, such control may be achieved by the photobiocatalytic systems described here or by using enzymatic donors of both electrons and H_2_O_2_ such as cellobiose dehydrogenase^47,48^. These latter set-ups allow the in situ and gradual production of both reducing equivalents and H_2_O_2_, offering thereby much greater control over the reaction.

### V-TiO_2_-promoted photobiocatalytic oxidation of cellulose

Turning our attention to the previously described photobiocatalytic system using V-TiO_2_ as photocatalyst and *Sc*AA10C as model enzyme^17^ (Fig. 1), we then investigated whether also this system could be based on the generation of ROS to fuel a peroxygenase mechanism, rather than on the generation of electrons to fuel a monooxygenase mechanism. Irradiation of V-TiO_2_ with visible light can lead to a variety of reactions, including production of H_2_O_2_ (Fig. 1; see ref. ^49^), but the V-TiO_2_/light system was originally thought to work by providing the LPMO with electrons analogous to reductants such as AscA.

**Fig. 8:**
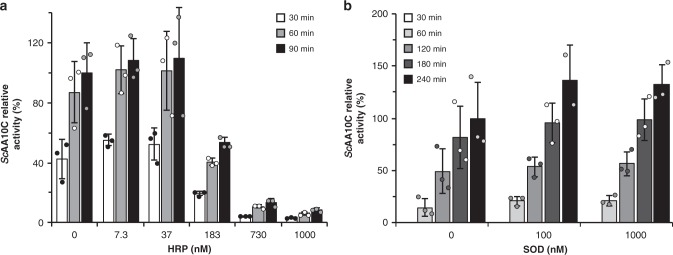
Probing the underlying role of reactive oxygen species in the light/V-TiO_2_/LPMO system. The graphs show time-courses for the release of aldonic acid products from Avicel (10 g L^−1^) by *Sc*AA10C (1 µM) in the presence of varying amounts of **a** horseradish peroxidase (HRP) and **b** superoxide dismutase (SOD). All reactions were carried out in sodium phosphate buffer (50 mM, pH 7.0) at 40 °C, under magnetic stirring and were fueled by a suspension of V-TiO_2_ (10 g L^−1^) exposed to visible light (*I* = 25% *I*_max_, approx. 42 W cm^−2^). Activities are expressed relatively (in %) to the quantity of products measured at the last time point of the reference reaction (i.e., without HRP or SOD). Before product quantification, solubilized cello-oligosaccharides were hydrolyzed by *Tf*Cel5A, to convert the LPMO products to a mixture of only two oxidized products with a degree of polymerization of 2 and 3 [GlcGlc1A, (Glc)_2_Glc1A], the amounts of which were summed up to yield the concentration of oxidized sites. Error bars show ± s.d. (*n* = 3, independent experiments). Control reactions in the dark without addition of HRP and SOD did not yield products. Note that the original work on the light/V-TiO_2_ system^17^ includes control reactions similar to those shown in Supplementary Fig. 4, which confirm that product formation depends on the enzyme and is not due to enzyme-independent reactions. Source data are provided as a Source Data file.

The possible involvement of ROS was investigated by analyzing whether the addition of a peroxidase to the system would influence V-TiO_2_/light-driven LPMO-catalyzed oxidation of cellulose (Fig. 8). In accordance with previous observations for standard LPMO reactions with the use of AscA or cellobiose dehydrogenase as source of reducing equivalents^22^, V-TiO_2_/light driven activity of *Sc*AA10C was increasingly inhibited when increasing the quantity of peroxidase (Fig. 8a). This result indicates that H_2_O_2_ is indeed generated in this photobiocatalytic system and sustains LPMO catalysis.

In our initial study, we observed low apparent H_2_O_2_ levels in reactions with LPMO^17^. With hindsight, we can conclude that H_2_O_2_ generated in these reactions was consumed by *Sc*AA10C. Given the importance of H_2_O_2_ in *Sc*AA10C catalysis and the occurrence of competing H_2_O_2_-consuming side-reactions at the V-TiO_2_ surface (e.g., photodecomposition^50^), it is not certain that this photocatalyst is ideal for promoting LPMO activity. The photoreactivity of metal-doped TiO_2_ depends on the nature and abundance of the metal used for doping^51^ and it is possible that other TiO_2_-based catalysts could be more suitable for light-driven LPMO catalysis. On this note, a 2018 study showed that a gold-coated TiO_2_ photocatalyst can fuel the (H_2_O_2_-consuming) unspecific peroxygenase from *Agrocybe aegerita* to hydroxylate aliphatic and aromatic compounds^52^. The use of alternative tailor-made photocatalysts designed for efficient H_2_O_2_ generation^53,54^ to drive LPMO reactions probably represents an interesting avenue of investigation.

Experiments with SOD added to the reaction (Fig. 8b) indicated that free superoxide likely is not generated in the V-TiO_2_/light/LPMO system, suggesting that the LPMO is reduced by reducing equivalents generated at the V-TiO_2_ surface. Accordingly, we have previously shown that light-exposed V-TiO_2_ reduces *Sc*AA10C under anaerobic conditions^17^. Of note, however, the rate-limiting step in this photobiocatalytic system likely resides in the thermodynamically challenging oxidation of water (*E*_0_^ox^ = −1.23 V vs. SHE for H_2_O/O_2_)^17,55^. Thus, if an O_2_^•−^ intermediate was formed, its accelerated conversion to H_2_O_2_ by SOD, may not be reflected in an increased LPMO rate, since none of the downstream reactions (relative to water oxidation) are rate-limiting. It is therefore difficult to conclude whether or not O_2_^•−^ is produced, although the two-electron reduction of O_2_ to H_2_O_2_ is thermodynamically much more likely than the single electron reduction to O_2_^•−^ (Supplementary Fig. 6).

### Light-driven activity of other LPMOs

Earlier work has shown that the V-TiO_2_/light system can drive multiple LPMOs, belonging to different families and acting on different substrates. The functionality of this system was demonstrated for a bacterial, C1-oxidizing, chitin-active AA10 LPMO (*Sm*AA10A)^2^, a fungal, C1-oxidizing, cellulose-active AA9 LPMO (*Pc*AA9D)^56^ and a bacterial, C1/C4 oxidizing, cellulose-active AA10 LPMO (*Sc*AA10B)^38^ (Fig. S3 in Bissaro et al.^17^).

To demonstrate the general applicability of the Chl/light system, we analyzed activity of *Sm*AA10A on chitin as well as the cellulose-oxidizing activity of a C1-oxidizing AA9 LPMO from the fungus *Neurospora crassa* (*Nc*AA9F or NCU03328)^57^. Supplementary Fig. 10 shows that both enzymes can be fueled by the Chl/light system, leading to chitin and cellulose oxidation, respectively. Of note, while showing the general applicability of the Chl/light system, these additional analyses showed that different LPMOs respond differently: while *Sm*AA10 performed better with Chl/light compared to AscA, *Nc*AA9F performed relatively poorly when driven by Chl/light and was rapidly inactivated. Differences in the way LPMOs respond to reductants and H_2_O_2_ are commonly observed^8,22,58^ and deserve further attention in future research.

The data presented in this study pinpoint several complications related to interpreting the outcome of LPMO reactions and provide insight into light-driven LPMO catalysis. One technical challenge concerns the measuring of actual H_2_O_2_ production rates by a given system in which H_2_O_2_ is the final product (i.e., not immediately used by an enzyme such as an LPMO). Our study also shows the intricacy of interpreting the effects of accessory enzymes such as catalase and SOD and demonstrates the absolute need for analyzing progress curves, rather than relying on single time point measurements of product formation. SOD accelerates the conversion of O_2_^•−^ into H_2_O_2_, which increases the LPMO initial rate but also leads to more rapid inactivation of the LPMO. On the other hand, catalase, by converting H_2_O_2_ into O_2_ and H_2_O, prevents accumulation of excess H_2_O_2_ and thus reduces LPMO inactivation. It is worth noting the linear progress curve that is obtained when using catalase in the light-driven reactions displayed in Fig. 2d. All in all, the present data clearly show that LPMOs use H_2_O_2_ and that controlling H_2_O_2_ levels is key to optimizing LPMO catalysis.

The set of experiments presented here demonstrates that when exposed to light, Chl can reduce O_2_ to O_2_^•−^ leading to H_2_O_2_ production, via either spontaneous disproportionation or chemical reduction (e.g., by AscA). H_2_O_2_ production can be regulated by light intensity but also by metalation of the porphyrin ring, which tunes the photochemical properties of the pigment. Although photo-excitation of the pigment is clearly needed to fuel LPMO activity, we were not able to discriminate whether reduction of the LPMO occurs via direct electron transfer from photo-excited Chl or via superoxide. Importantly, studies of a completely different system for light-driven driven LPMO catalysis, based on using V-TiO_2_ particles, showed that also in this case light-driven oxidation of cellulose by the LPMO entails a peroxygenation reaction. Of note, the earlier study on driving LPMO reactions with the V-TiO_2_/light system^17^ showed that LPMO activity can be controlled by switching the light on or off and that the system also works when using regular sunlight.

Most importantly, our data show that these LPMO-catalyzed peroxygenation reactions can be fueled by light only and that other sources of energy, such as reducing equivalents used in standard LPMO reactions and in earlier work on light-driven LPMO catalysis, are not required. The use of light allows tunable in situ generation of H_2_O_2_ which then fuels polysaccharide oxidation at rates that are higher than those reported when LPMOs were still believed to act as monooxygenases only. Considering the large current interest in copper catalysts (e.g., Snyder et al.^59^), we expect that the present findings will have implications beyond processing of biomass.

## Methods

### Materials

Chemicals and enzymes were purchased from Sigma-Aldrich unless indicated otherwise. The crystalline cellulose used was Avicel® PH-101 (~50 µM particles). The superoxide dismutase (SOD, recombinantly expressed in *E. coli*) was stored (100 µM, eq. 1.63 mg mL^−1^) in sodium phosphate buffer (100 mM, pH 7.5) at 4 °C. The peroxidase from horseradish (HRP, type II) was stored (0.5 mg mL^−1^, eq. 100 U mL^−1^) in sodium phosphate buffer (50 mM, pH 6.0) at 4 °C. The catalase katE from *Streptomyces sirex* (recombinantly expressed in *E. coli*) was produced in-house and stored (1.8 mg mL^−1^) in Tris-HCl buffer (50 mM, pH 8.0) at −20 °C. Ascorbic acid (100 mM) and Amplex red (10 mM) stock solutions were prepared in water and DMSO respectively, aliquoted, stored at −20 °C, and thawed in the dark for 10 min just before use. The V-TiO_2_ powder was kindly provided by Dr. Frank Hollmann (Delft University, Netherlands) and prepared according to a previously described protocol^60^. According to literature, the Chl purchased from Sigma consists of about 72% Cu(II)-chlorin e_6_ and 10% Cu(II)-isochlorin e_4_^37^. Chlorin e_6_ was purchased from Frontier Scientific (Logan, Utah, USA). Copper-reconstituted chlorin e_6_ was prepared by incubating chlorin e_6_ with 0.9 molar equivalent of CuSO_4_ for 30 min at 4 °C in deionized Milli-Q water.

### Production and purification of recombinant LPMOs

The recombinant AA10 LPMO from *Streptomyces coelicolor* (*Sc*AA10C) was produced and purified according to previously described protocols^1,38^. Note that *Sc*AA10C refers to the wild-type full-length enzyme, which comprises an AA10 domain connected via a linker to a CBM2 domain (UniProt Q9RJY2). *Sc*AA10C was prepared and stored in sodium phosphate (50 mM, pH 6.0), copper-saturated with Cu(II)SO_4_ and desalted (PD MidiTrap G-25, GE Healthcare) before use^10^. LPMOs from *Serratia marcescens* (*Sm*AA10A) and *Neurospora crassa* (*Nc*AA9F or NCU03328) were produced and purified as previously described^1,57^ and copper saturated in the same way as for *Sc*AA10C, except that the buffer was Bis-Tris pH 6.5 and pH 6.0, respectively.

### Standard photobiocatalytic reaction conditions

The reactor was a cylindrical glass vial (1.1 mL) with conical bottom (Thermo Scientific) and the reaction volume was 500 µL. Typical reactions were carried out as follows: the enzyme (0.5 µM) and Avicel (10 g L^−1^) were mixed in sodium phosphate buffer (50 mM final concentration after all additions; pH 7.0 or 6.0 for Chl and V-TiO_2_ studies, respectively) followed by incubation at 40 °C under magnetic stirring during 20 min. Photobiocatalytic reactions contained either chlorophyllin or chlorin e_6_ (500 µM, unless stated otherwise) or V-TiO_2_ (5 mg mL^−1^) as a light harvester. The reaction was initiated by adding ascorbic acid (to a final concentration of 1 mM, unless stated otherwise), and/or turning on the light (*I* = 25% *I*_max_, eq. to 42 W cm^−2,^^17^, unless stated otherwise). At regular intervals, 55 µL samples were taken from the reaction mixtures and soluble fractions were immediately separated from the insoluble substrate by filtration using a 96-well filter plate (Millipore) operated with a vacuum manifold. When it was needed to also measure H_2_O_2_ in the reaction mixture, the 55 µL sample was mixed with 55 µL of NaOAc buffer (50 mM, pH 4.5) before filtration (see below). By separating soluble and insoluble fractions, LPMO activity is stopped, as the LPMO used in this study does not oxidize soluble cello-oligosaccharides. Filtered samples were frozen (−20 °C) prior to further analysis. Prior to product quantification, 30 µL of sample was mixed with 30 µL of a solution of endoglucanase Cel5A from *Thermobifida fusca* (*Tf*Cel5A, 2 µM in the premix) prepared in Bis-Tris buffer (25 mM, pH 6.0), followed by incubation overnight at 40 °C to convert the solubilized cello-oligosaccharides to a mixture of glucose, cellobiose and C1-oxidized products with a degree of polymerization of 2 and 3 [GlcGlc1A, (Glc)2Glc1A]. For chlorin e_6,_ the 50 µL reaction mixture was mixed with 50 µL 0.2 M CH_3_COONa pH 4.0 prior to filtration to ensure chlorin e_6_ precipitation on the filters. *Tf*Cel5A for treatment of samples containing chlorin e_6_ was prepared in 0.2 M CH_3_COONa pH 6.5 (to increase pH for efficient solubilization of cello-oligosaccharides) and added to the filtrate as described above.

### Analysis of reaction products

For qualitative analysis, samples were analysed by MALDI-TOF MS, as previously described^2^. For quantitative analysis, cello-oligosaccharides (native and oxidized) were separated by high performance anion exchange chromatography (HPAEC) and monitored by pulsed amperometric detection (PAD) using a Dionex Bio-LC equipped with a CarboPac PA1 column as previously described^61^. Chromatograms were recorded and analyzed using Chromeleon 7.0 software. Oxidized dimers and trimers were quantified using GlcGlc1A and (Glc)_2_Glc1A standards obtained by incubating (40 °C, 1000 rpm) cellobiose (2 mM) or cellotriose (2 mM) with the cellobiose dehydrogenase from *Myriococcum thermophilum* (*Mt*CDH,^62^ 2 μM, 3 successive additions every 24 h to obtain maximum conversion of 95%). Chito-oligosaccharides resulting from the action of *Sm*AA10A on β-chitin were analyzed using a Dionex Ultimate 3000 UHPLC system equipped with a Rezex RFQ-Fast acid H^+^ (8%) 7.8 × 100 mm column as previously described^45^. The elution of chitooligosaccharides was monitored using a UV detector (194 nm). Prior to analysis of solubilized mixtures of chitooligosaccharides, these chitooligosaccharides were hydrolyzed with a chitobiase, *Sm*GH20, from *S. marcescens* (1 µM final concentration) yielding chitobionic acid as the only oxidized product^10^.

### H_2_O_2_ production measurements

The method is adapted from a previously reported protocol^57^ with some modifications explained hereinafter. For each reaction (carried out as described above), 55 µL were sampled at regular intervals and mixed with 55 µL of NaOAc buffer (50 mM, pH 4.5) before filtration as described above. Notably, the decrease in pH obtained by the addition of NaOAc makes chlorophyllin insoluble, meaning that this compound (if present) was removed from the solution during the filtration step, leading to a transparent and stable filtrate usable for colorimetric analysis. Thirty microliter of each filtrate was saved for analysis of oxidized products analysis when applicable (cf above). For reactions with chlorin e_6_ (metallated and non-metallated), a slightly different treatment was applied to remove the pigment: both chlorin e_6_ compounds were diluted in a 1:1 ratio with sodium acetate (200 mM, pH 4.0).

To determine the H_2_O_2_ concentration (in all reactions devoid of pigment and for those containing Chl), 50 µL of the filtrate (or dilutions of it, if necessary) were mixed with 50 µL of a premix composed of HRP (10 U/mL) and Amplex Red (200 µM, 2% DMSO, in the premix) in sodium phosphate buffer (50 mM, pH 7.5). For chlorin e_6_, 10 µL of the filtrated sample was combined with 90 µL premix of HRP and Amplex Red (10 U/mL and 200 µM final concentrations respectively), while for chlorin e_6_-Cu(II) 50 µL filtrate was combined with 50 µL HRP and Amplex Red premix (10 U/mL and 200 µM final concentrations, respectively). The buffer used was sodium phosphate (250 mM, pH 7.0) to ensure proper buffering. The reaction mixture (100 µL) was incubated in a 96-well microtiter plate during 10 min before recording the absorbance at 540 nm. For each set of measurements, a blank and H_2_O_2_ standards were prepared alike the corresponding samples and subjected to the same buffer treatment. Also, an average background control was included to account for the absorbance coming from residual soluble chlorophyllin (small quantities were observed for time points beyond 4 h). To generate this background control, 18 µL portions of the filtrates from each sample of a triplicate chlorophyllin-containing reaction were pooled. 50 µL of this 54 µL pool (or a dilution equivalent to the one used for the reaction containing Amplex red) was mixed with 50 µL of a premix made of HRP (10 U/mL) and DMSO (2% in the premix) in sodium phosphate buffer (50 mM, pH 7.5) (i.e., the same premix as previously described but without Amplex red). The difference (if any) between this background control and the blank sample was used to correct for absorbance generated by residual chlorophyllin.

### Verification of superoxide dismutase (SOD) activity

SOD activity was verified according to a published protocol^63^. In brief, a stock solution of pyrogallol (15 mM) was prepared in 10 mM HCl; the tube was wrapped in aluminum foil and kept on ice. Prior to each measurement, the background absorbance (*A*_325_ nm) of 50 mM Tris-HCl pH 8.0 was monitored and allowed to stabilize. For the reference reaction, pyrogallol was added to a final concentration of 200 µM and the *A*_325_ nm was measured every 10 s for 5 min. For the superoxide dismutase (SOD) reactions, SOD was quickly added after the pyrogallol to final concentrations of 10, 100, 500, and 1000 nM. The solution was pipetted up and down for mixing and the *A*_325_ nm was recorded every 10 s for 5 min with the same conditions as for the reference reaction i.e., 200 µM pyrogallol in 50 mM Tris-HCl pH 8.0.

### Voltammetric measurements

Stock solutions of chlorophyllin and chlorin e_6_ (5 mM) were prepared fresh each day in deionized Milli-Q water. These were protected from light in aluminum foil and kept on ice. Prior to voltammetric measurements, Chl or chlorin e_6_ was diluted ten-fold (500 µM) in potassium phosphate buffer (100 mM, pH 7.0) supplemented with KCl as supporting electrolyte (100 mM). The voltammetric measurements were carried out using an MCS-200 potentiostat (BioLogic, France) and EC-lab software (BioLogic, France). The voltammograms were recorded using a three-electrode system (eDAQ, Australia) with a glassy-carbon electrode (GCE) of 1.0 mm diameter as the working electrode, an Ag/AgCl (3.4 M KCl) electrode as a reference electrode, and a platinum covered titanium wire as a counter electrode. The glassy-carbon electrode was cleaned before each measurement. It was rinsed with deionized Milli-Q water prior to polishing using a 0.05 µm Alumina polishing slurry (eDAQ, Australia) on a wet polishing cloth. Then, the electrode was rinsed with deionized Milli-Q water before and after a 30 s sonication step. The solutions were degassed by nitrogen sparging before the electrochemical measurements, and a nitrogen blanket over the solution was maintained during the experiments. All experiments were performed at room temperature in the dark. To obtain the square-wave voltammograms, we used the same parameters as described in ref. ^37^. The pulse height (amplitude) was 50 mV, the step height (potential increment) was 2 mV and the pulse width (frequency) was 0.4 ms (1250 Hz), which corresponds to a scan rate of 2500 mV s^−1^. The Ag/AgCl reference electrode was calibrated each day by cyclic voltammetry using potassium ferricyanide [K_3_Fe(CN)_6_] (1 mM) in potassium phosphate buffer (100 mM pH 7.0) supplemented with KCl as supporting electrolyte (100 mM). All the potentials reported were adjusted to refer to the standard hydrogen electrode (SHE) using the conversion factor *E*_Ag/AgCl_ vs. SHE = 222.49 mV for our reference electrode^64^. Of note, some previously determined potentials used in this paper were reported relative to the NHE; the difference between the two is that the SHE is appr. 6 mV lower than the NHE^65^.

### Fluorescence measurements

Fluorescence signals were recorded using a Cary Eclipse Fluorescence spectrophotometer (Agilent Technologies) using a 2 mL quartz cuvette (Hellma Analytics, 101-QS). Data acquisition was performed at room temperature with a PMT detector voltage of 600 V. Excitation and emission spectra were acquired at respective maximum emitting and exciting wavelengths, determined iteratively and shown in Supplementary Fig. 1.

### Reporting summary

Further information on research design is available in the Nature Research Reporting Summary linked to this article.

## Supplementary information

Supplementary Information

Reporting Summary

## Data Availability

Data supporting the findings of this work are available within the paper and its Supplementary Information files and from the corresponding author upon reasonable request. A reporting summary for this Article is available as a Supplementary Information file. The source data underlying Figs. 2, 3, 4, 5, 6, 7, 8 as well as Supplementary Figs. 3, 5, 7, 8, 9, 10, 11, and Supplementary Table 1 are available as a Source Data file.

## Source Data

Source Data
