# Supplementary material for: Controlled depolymerization of cellulose by light-driven lytic polysaccharide oxygenases
Source: Nat Commun. 2020 Feb 14;11:890. doi: 10.1038/s41467-020-14744-9 (PMC7021734; doi:10.1038/s41467-020-14744-9)
Supplement: Supplementary file 1 — Supplementary Information [file 41467_2020_14744_MOESM1_ESM.pdf]

SUPPLEMENTARY INFORMATION FOR

Controlled Depolymerization of Cellulose by Light-Driven Lytic Polysaccharide Oxygenases

Bastien Bissaro[1,2,†], Eirik Kommedal[1,†], Åsmund K. Røhr[1] and Vincent G.H. Eijsink[1*]

[1]Faculty of Chemistry, Biotechnology and Food Science, Norwegian University of Life Sciences (NMBU), N-1432 Ås, Norway

[2]INRAE, Aix Marseille University, UMR1163 Biodiversité et Biotechnologie Fongiques, 13009, Marseille, France

† These authors contributed equally
* corresponding author: vincent.eijsink@nmbu.no

This file includes:

Supplementary Figures
Supplementary Tables
Supplementary References

Supplementary Figures

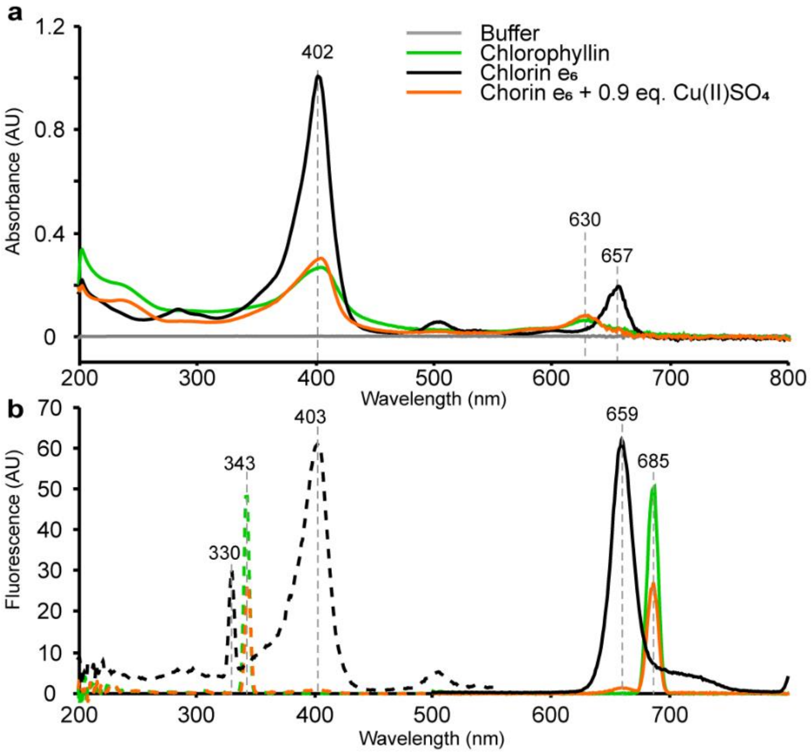

Supplementary Figure 1. Absorbance and fluorescence spectral characterization of chlorophyllin and chlorin e6. (a) Absorbance spectra of chlorophyllin (12.5 µM; green line), copper-free chlorin e_6 (12.5 µM; black line) and chlorin e_6 complemented with 0.9 eq. of $Cu(II)SO_4$ (12.5 µM; orange line). The Soret (λ^{max} = 402 nm) and Q_y (λ^{max} = 630 nm) bands are characteristic of porphyrin rings; depletion of copper gave an expected[1] red shift of the Q_y band for chlorin e_6 (λ^{max} = 657 nm). **(b)** Emission (solid lines) and excitation (dotted lines) fluorescence spectra of chlorophyllin (5 µM; Ex343/Em685, green lines), chlorin e_6 (125 nM; Ex403/Em659, black lines) and chlorin e_6 complemented with 0.9 eq. of $Cu(II)SO_4$ (5 µM; Ex343/Em685, orange lines). The spectra show that copper binding quenches the fluorescence signal and induces a shift in the optimal excitation and emission wavelengths, which is consistent with changes in electronic properties. All solutions were prepared in potassium phosphate buffer (100 mM, pH 7.0) with 100 mM KCl and flushed with N_2.

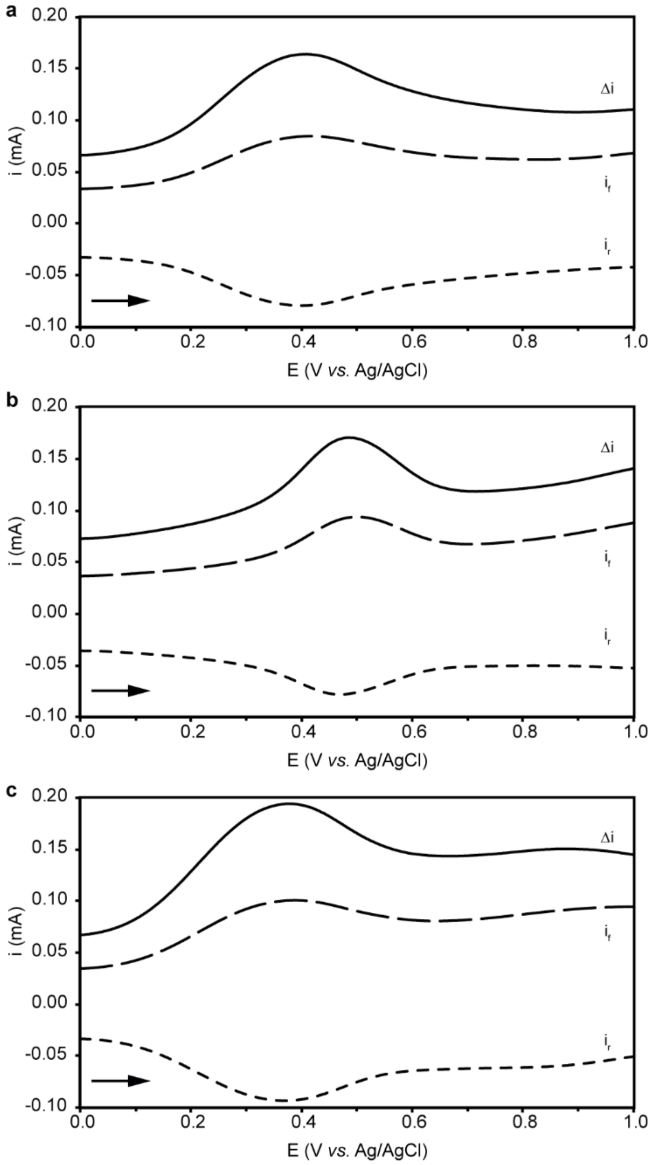

Supplementary Figure 2. Square-wave voltammograms for chlorophyllin compounds. The panels show the square-wave voltammograms obtained for **(a)** Chl (500 µM), **(b)** chlorin e_6 (500 µM), and **(c)** chlorin e_6-Cu(II) (500 µM). All chlorophyllin compounds were prepared in potassium phosphate buffer (100 mM, pH 7.0) with KCl (100 mM) as supporting electrolyte in anaerobic conditions and protected from light using aluminum foil. The amplitude was 50 mV, the potential increment 2 mV and the frequency 1250 Hz. All potentials were recorded *versus* an Ag/AgCl reference electrode and were adjusted to refer to the standard hydrogen electrode (SHE) using $E_{Ag/AgCl\ vs.\ SHE}$ = 222.49 mV[2].

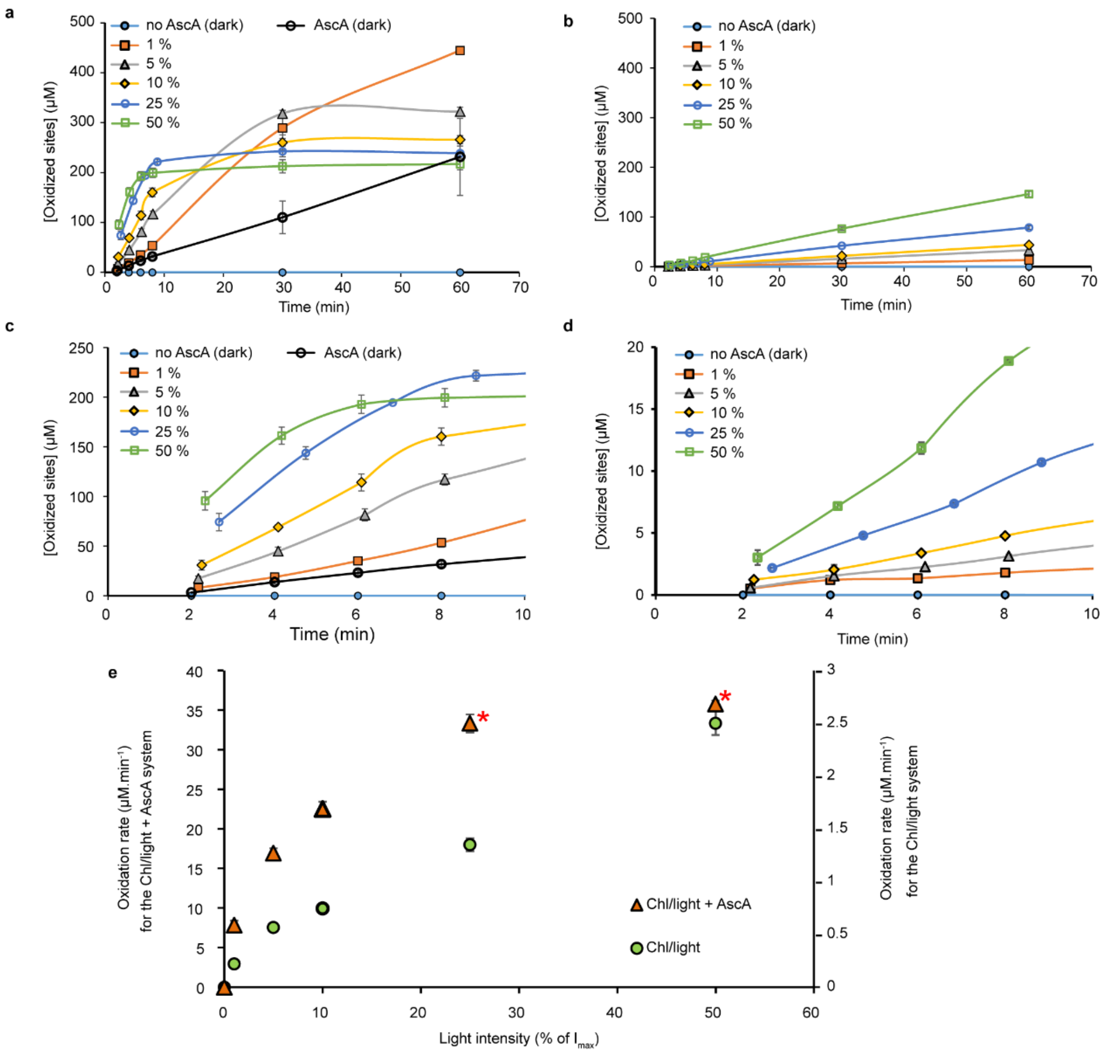

Supplementary Figure 3. The effect of light intensity on *Sc*AA10C-catalyzed cellulose oxidation fueled by Chl/light. *Sc*AA10C reactions in **(a)** the presence of AscA (1 mM) and **(b)** the absence of AscA. Panels **(c)** and **(d)** show enlargements of parts of panels **(a)** and **(b)**, respectively. Panels **(a)** and **(c)** also show data for a reaction with only AscA (1 mM). Panel **(e)** shows the approximate initial oxidation rates (expressed as µM of oxidized sites/min) as a function of light intensity for both systems. Note the different Y-axes; the system with AscA is much faster. For reactions carried out in the presence of AscA and with a light intensity of I = 25 or 50 % of I_{max} the reaction was so fast that the rate (derived from the 2 first time points) is probably underestimated (red stars). Reactions were carried out with 0.5 µM *Sc*AA10C in sodium phosphate buffer (50 mM, pH 7.0) at 40 °C, under magnetic stirring and exposed to visible light (I = 0-50% I_{max}, approx. 0-84 W.cm^{-2}). Before product quantification, solubilized cello-oligosaccharides were hydrolyzed by *Tf*Cel5A, to convert the LPMO products to a mixture of only two oxidized products with a degree of polymerization of 2 and 3 [GlcGlc1A, $(Glc)_2$Glc1A], the amounts of which were summed up to yield the concentration of oxidized sites. Error bars show ± s.d. (n = 2, independent experiments). Source data are provided as a Source Data file.

Of note, the light intensities used in our study are higher than those used by Cannella and colleagues[10]. This Figure (and corresponding Figure 3 in the main text) show that it is possible to use lower light intensities, but the reactions then proceed slower.

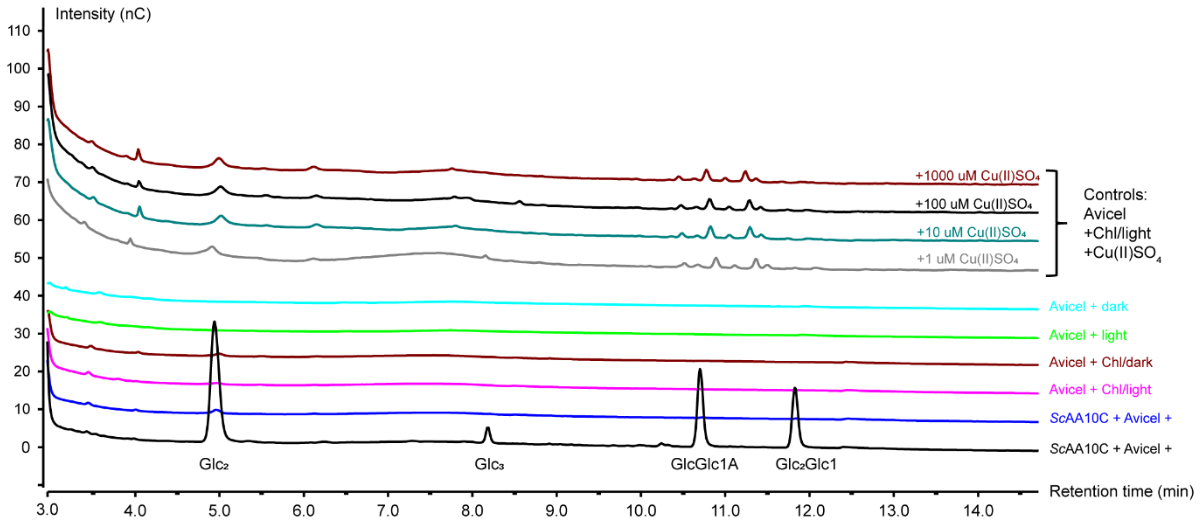

Supplementary Figure 4. Control reactions to check for the occurrence of copper-catalyzed Fenton-type chemistry. The picture shows chromatographic product profiles obtained in various control reactions. In the upper four reactions, the enzyme, *Sc*AA10C, was replaced by different concentrations of Cu(II)SO_4 (0 – 1000 µM) whereas the positive control reaction at the bottom, contained 1 µM of *Sc*AA10C. Product profiles obtained in additional control reactions (no light, no enzyme or copper, no chlorophyllin) are also shown. Reactions were carried out in sodium phosphate buffer (50 mM, pH 7.0) at 40 °C, under magnetic stirring with Avicel (10 g.L^{-1}) and Chl (500 µM) and exposed to visible light (I = 25 % I_{max}, approx. 42 W.cm^{-2}), as indicated. Before chromatographic analysis, the samples were treated with a cellulase, *Tf*Cel5A, to convert soluble products to a mixture of only two oxidized products with a degree of polymerization of 2 and 3 [GlcGlc1A, $(Glc)_2$Glc1A]. The figure shows a superimposition of the resulting chromatograms. Minor amounts of non-specific products can be observed in the $CuSO_4$ controls, which are not observed in the LPMO-containing reactions, which only show the expected oxidized oligosaccharides. Two independent replicates of the experiment were carried out.

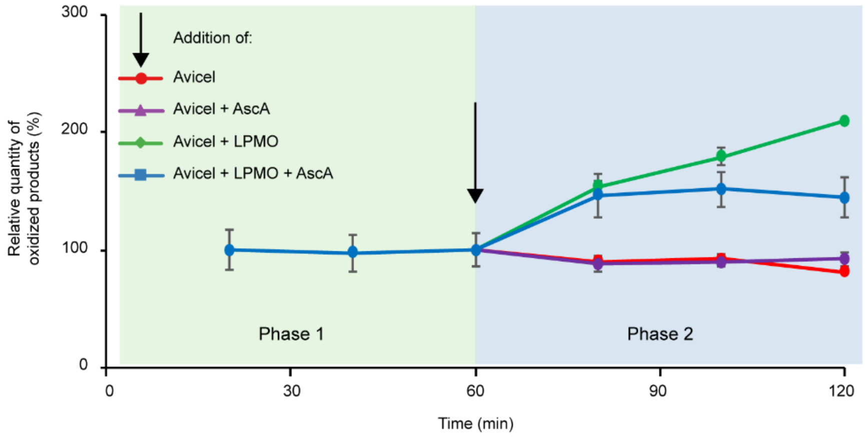

Supplementary Figure 5. Control reactions to check for inactivation of the LPMO during incubation with Chl/light and AscA. The figure shows time courses for the release of oxidized products from Avicel by *Sc*AA10C. Samples were withdrawn every 20 min and filtered to stop the reactions. In phase 1 (0-60 min), *Sc*AA10C (0.5 µM) was incubated with Avicel (5 $g.L^{-1}$) in presence of AscA (1 mM) and Chl (500 µM)/light. In phase 2 (60 – 120 min), subsequently to the 60 min sampling, the reactions were supplemented (black arrow) with fresh reaction components (given concentrations are final concentrations of the added material after mixing), as follows: Avicel (5 $g.L^{-1}$) (red circles); Avicel (5 $g.L^{-1}$) and AscA (1 mM) (purple triangles); Avicel (5 $g.L^{-1}$) and *Sc*AA10C (0.5 µM) (green diamonds); Avicel (5 $g.L^{-1}$), AscA (1 mM) and *Sc*AA10C (0.5 µM) (blue squares). To achieve this, the phase 1 mixtures (380 µL) were diluted with solutions containing Avicel (100 $g.L^{-1}$), AscA (100 mM) and/or *Sc*AA10C (25 µM) to obtain the concentrations given above. Reactions were carried out in sodium phosphate buffer (50 mM, pH 7.0) at 40 °C, under magnetic stirring and exposed to visible light ($I = 25$ % I_{max}, approx. 42 $W.cm^{-2}$). Before product quantitation, solubilized cello-oligosaccharides were hydrolyzed with *Tf*Cel5A, to convert the LPMO products to a mixture of only two oxidized products with a degree of polymerization of 2 and 3 [GlcGlc1A, $(Glc)_2$Glc1A], the amounts of which were analyzed by HPLC and summed up to yield the concentration of oxidized sites. The quantities of oxidized products are expressed as percentage of the quantity of products measured at 60 min. The dilution factor due to the addition of fresh reaction components in phase 2 was taken into account. Error bars show ± s.d. (n = 2, independent experiments). Source data are provided as a Source Data file.

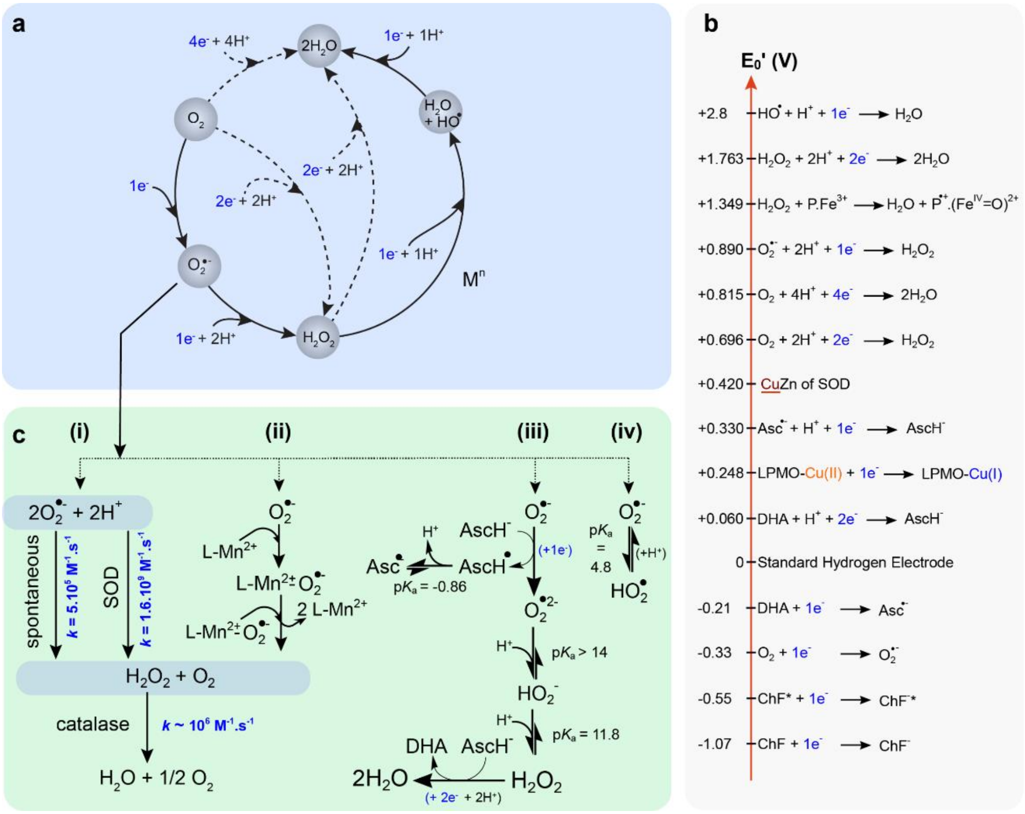

Supplementary Figure 6. Generation and fate of ROS. Panel **(a)** (blue frame) shows the reduction cycle for O_2 and derived ROS. Panel **(b)** (grey frame) shows potentials for reduction reactions potentially occurring in the systems under study. Single electron redox potentials of different ascorbic acid species at pH 7.0 were obtained from Figure 8 of Iyanagi et al.[3]. Reduction potential values (at pH 7.0, *vs* SHE) for O_2 and ROS couples were retrieved from Wood et al.[4] and those for the oxidized state (ChF) and the photoexcited triplet state (ChF*) of chlorophyll *a* from Seely et al.[5]. Panel **(c)** shows different pathways for the conversion of $O_2^{\bullet-}$ to other ROS. $O_2^{\bullet-}$ can be disproportionated to H_2O_2 spontaneously or enzymatically (by SOD) (**pathway i**) but also by manganese-binding complexes (e.g. L=phosphate[6]) (**pathway ii**). $O_2^{\bullet-}$ can also be further reduced (e.g. by AscA) to a peroxide ion ($O_2^{\bullet 2-}$), which is in fast equilibrium with H_2O_2 in non-alkaline conditions (**pathway iii**). In agreement with redox potentials (panel **b**), it has been shown in the literature that the reduction of $O_2^{\bullet-}$ to H_2O_2 can be accelerated by AscA[7,8], as also observed here (**Figure 3c**). In acidic conditions, $O_2^{\bullet-}$ can be protonated (pK_a = 4.8) yielding a hydroperoxyl radical (**pathway iv**). H_2O_2 can be further reduced to H_2O by reductants (e.g. by AscA) or enzymes (e.g. by catalase), as indicated in the Figure. Rate constants reported in panel **(c)** were retrieved from Augusto et al.[9]. DHA, dehydroascorbic acid; M^n, reduced free metal.

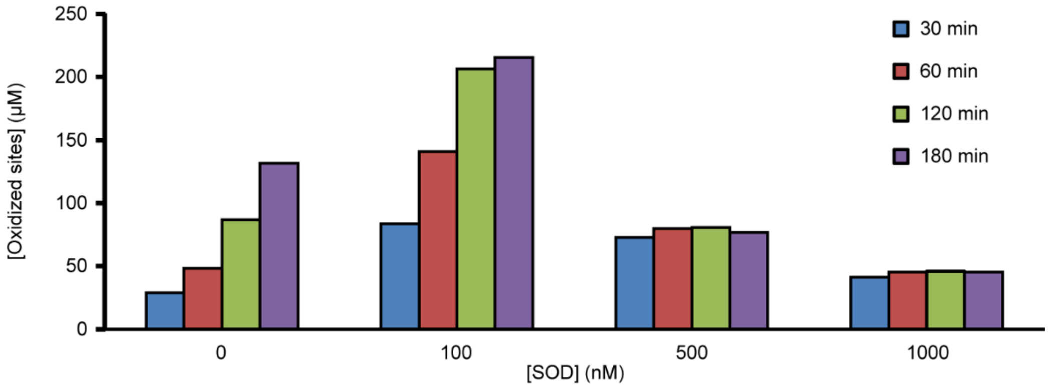

Supplementary Figure 7. The impact of SOD on *Sc*AA10C-catalyzed oxidation of Avicel fueled by the Chl/light system. The figure shows time courses for the release of aldonic acid products from Avicel (10 g.L^{-1}) by *Sc*AA10C (0.5 µM) fueled by Chl (500 µM)/light, in reaction mixtures supplemented with different concentrations of SOD (0-1000 nM) and with no AscA. Reactions were carried out in sodium phosphate buffer (50 mM, pH 7.0) at 40 °C, under magnetic stirring and exposed to visible light (I = 25% I_{max}, approx. 42 W.cm^{-2}). Before product quantification, solubilized cello-oligosaccharides were hydrolyzed by *Tf*Cel5A, to convert the LPMO products to a mixture of only two oxidized products with a degree of polymerization of 2 and 3 [GlcGlc1A, $(Glc)_2$Glc1A], the amounts of which were summed up to yield the concentration of oxidized sites. This figure shows the trade-off between SOD-catalyzed increased generation of H_2O_2 in the reaction mixtures, leading to higher LPMO catalytic rates, and enzyme inactivation. 100 nM SOD gives a higher initial LPMO rate, higher yields after 180 min but also signs of faster inactivation, i.e. slowing down of product formation in the later phase of the reaction (compared to the reaction without SOD). At higher SOD concentrations (i.e. higher levels of generated H_2O_2), the LPMO is inactivated very rapidly, so fast in fact that the product yield after 30 min does not reach the level reached at 100 nM SOD. This screening experiment was carried out once (n =1). Source data are provided as a Source Data file.

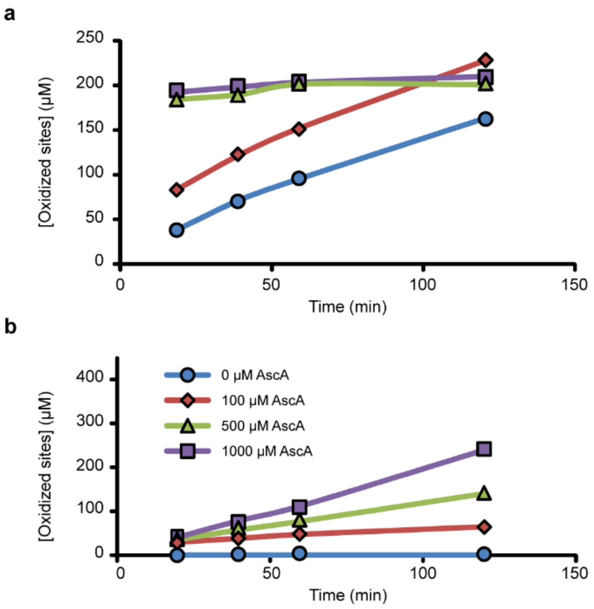

Supplementary Figure 8. The impact of the ratio between Chl and AscA on LPMO activity and stability. The figures show time courses for the release of oxidized products from Avicel (10 $g.L^{-1}$) by *Sc*AA10C (0.5 µM) in combination with different doses (0-1000 µM) of AscA **(a)** with (500 µM) and **(b)** without Chl. Reactions were carried out in sodium phosphate buffer (50 mM, pH 7.0) at 40 °C, under magnetic stirring and exposed to visible light (I = 25% I_{max}, approx. 42 $W.cm^{-2}$). Before product quantification, solubilized cello-oligosaccharides were hydrolyzed by *Tf*Cel5A, to convert the LPMO products to a mixture of only two oxidized products with a degree of polymerization of 2 and 3 [GlcGlc1A, $(Glc)_2$Glc1A], the amounts of which were summed up to yield the concentration of oxidized sites. This figure shows that, as expected, AscA alone can drive the LPMO action in a concentration-dependent manner. The Chl/light system works without AscA, but becomes very effective in the presence of higher AscA concentrations. However, these latter conditions lead to rapid inactivation of the LPMO. This screening experiment was carried out once (n =1). Source data are provided as a Source Data file.

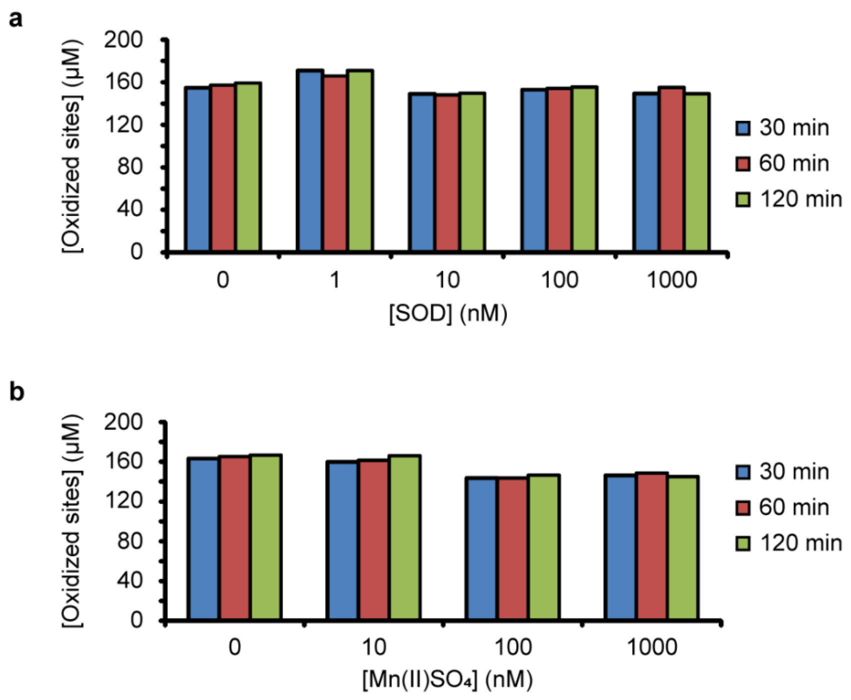

Supplementary Figure 9. The impact of SOD or Mn(II)SO$_4$ on the rate of light-induced LPMO-catalyzed cellulose degradation. The graphs show time courses for the release of aldonic acid products from Avicel (10 g.L^{-1}) by *Sc*AA10C (0.5 µM) fueled by the Chl (500 µM)/light + AscA (1 mM) system and supplemented with different doses of **(a)** SOD (0-1000 µM) or **(b)** Mn(II)SO$_4$, which remove superoxide enzymatically and chemically, respectively (carbonate or phosphate manganese complexes are described as SOD equivalents, see pathway (ii) in **Supplementary Figure 6**). Reactions (300 µL total volume) were carried out in sodium phosphate buffer (50 mM, pH 7.0) at 40 °C, under magnetic stirring and exposed to visible light (I = 25% I_{max}, approx. 42 W.cm^{-2}). Before product quantification, solubilized cello-oligosaccharides were hydrolyzed by *Tf*Cel5A, to convert the LPMO products to a mixture of only two oxidized products with a degree of polymerization of 2 and 3 [GlcGlc1A, (Glc)$_2$Glc1A], the amounts of which were summed up to yield the concentration of oxidized sites. The results show that at the first time point, the LPMO is inactivated and that addition of superoxide-removing agents does not change this. This screening experiment was carried out once (n =1). Source data are provided as a Source Data file.

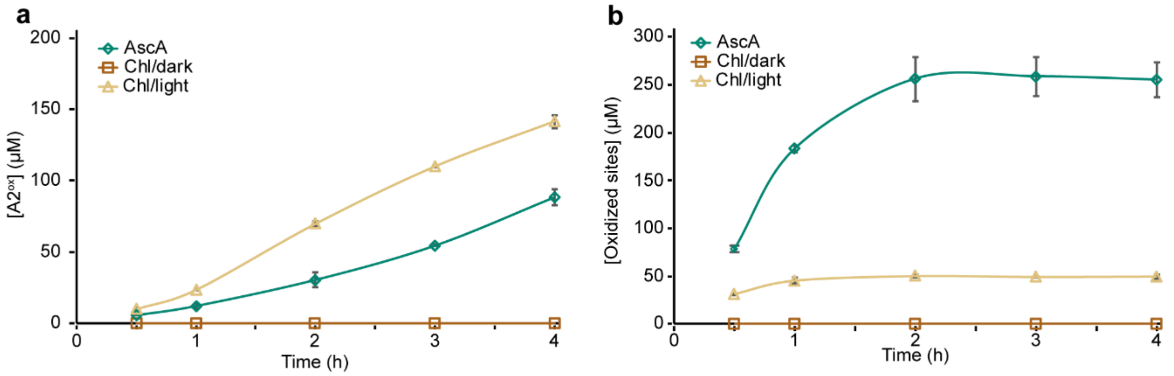

Supplementary Figure 10. Chl/light driven activity of other LPMOs. The figures show time courses for the release of oxidized products from **(a)** β-chitin (10 g.L^{-1}) by *Sm*AA10A (0.5 µM) and **(b)** Avicel (10 g.L^{-1}) by *Nc*AA9F (0.5 µM) in presence of AscA (1 mM), Chl (500 µM)/dark or Chl (500 µM)/light. The color code is provided in the figure. Reactions were carried out in sodium phosphate buffer (50 mM, pH 7.0) at 40 °C, under magnetic stirring and exposed to visible light (I = 25 % I_{max}, approx. 42 W.cm^{-2}). Before product quantitation, solubilized chito-oligosaccharides were hydrolyzed using a chitobiase, *Sm*GH20, to convert soluble LPMO products to chitobionic acid (A2^{ox}) and N-acetylglucosamine, after which the former was quantified by HPLC analysis. Solubilized cello-oligosaccharides were hydrolyzed using a cellulase, *Tf*Cel5A, to convert the LPMO products to a mixture of only two oxidized products with a degree of polymerization of 2 and 3 [GlcGlc1A, $(Glc)_2$Glc1A], the amounts of which were analyzed by HPLC summed up to yield the concentration of oxidized sites. Error bars show ± s.d. (n = 2, independent experiments). Source data are provided as a Source Data file.

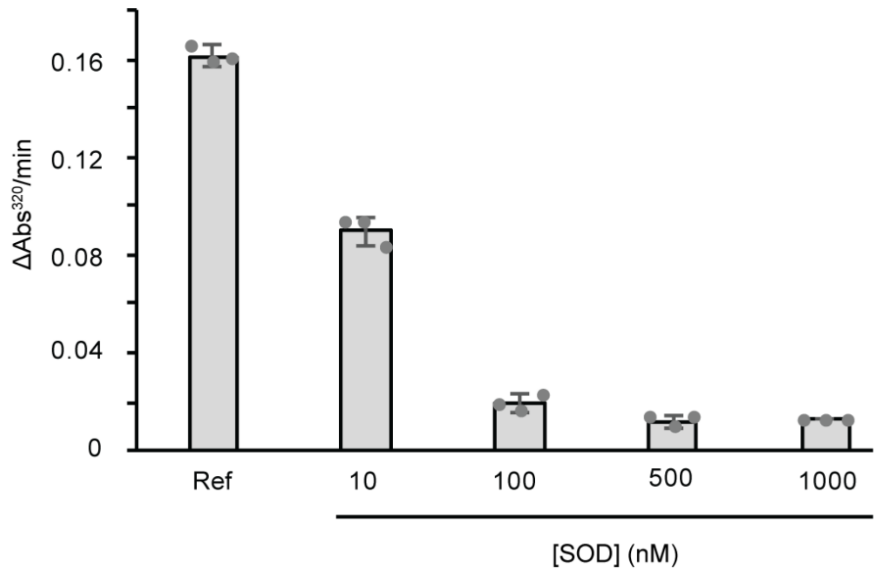

Supplementary Figure 11. Inhibition of pyrogallol auto-oxidation by SOD to verify SOD activity. The figure shows the rate of formation of purpurogallin, measured as the change in absorbance at 320 nm derived from a 5-min linear time-course. Purpurogallin is generated by auto-oxidation of pyrogallol and its formation is dependent on the availability of superoxide generated during the first step of the auto-oxidation process[11]. The reference reaction ("Ref") consists of pyrogallol (200 μM) in Tris-HCl buffer (50 mM, pH 8.0) and shows maximum auto-oxidation of pyrogallol and, thus, maximum formation of purpurogallin. Addition of different amounts of SOD (0-1000 nM) inhibits the formation of purpurogallin in a dose-dependent manner, which demonstrates that the SOD is active. All reactions were carried out at 25 °C. The reactions were initiated by the addition of pyrogallol and the absorbance was measured every 10 s for 5 min. The stock solution of pyrogallol was prepared in 10 mM HCl to prevent auto-oxidation. Error bars show ± s.d. (n = 3, independent experiments). Source data are provided as a Source Data file.

Supplementary Tables

Supplementary Table 1. Square-wave voltammetric peak potentials of chlorophyllin compounds[a].

Chl species	E_p (V vs. SHE)
Chlorophyllin	0.634 ± 0.004 V
Chlorin e_6	0.709 ± 0.003 V
Chlorin e_6-Cu(II)	0.601 ± 0.019 V

[a] Chl (500 µM), chlorin e_6 (500 µM), and chlorin e_6-Cu(II) (500 µM) were prepared in potassium phosphate buffer (100 mM, pH 7.0) with KCl (100 mM) as supporting electrolyte in anaerobic conditions and protected from light using aluminum foil. The amplitude was 50 mV, the potential increment 2 mV and the frequency 1250 Hz. All potentials were recorded *versus* an Ag/AgCl reference electrode and were adjusted to refer to the standard hydrogen electrode (SHE) using $E_{Ag/AgCl\ vs.\ SHE}$ = 222.49 mV[2]. The errors correspond to the standard deviation of three independent experiments. Source data are provided as a Source Data file.

Supplementary References

1. Gouterman, M. in *The Porphyrins, Volume III: Physical Chemistry* (ed. Dolphin, D.) (Academic Press, San Diego, 1978).
2. Spitzer, P. *et al.* in *Handbook of Reference Electrodes* (ed. Inzelt G., Lewenstam A., Scholz F.) (Springer-Verlag Berlin and Heidelberg GmbH & Co. KG, Berlin, 2013).
3. Iyanagi, T., Yamazaki, I. & Anan, K. F. One-electron oxidation-reduction properties of ascorbic acid. *Biochim. Biophys. Acta* **806**, 255–261 (1985).
4. Wood, P. M. The potential diagram for oxygen at pH 7. *Biochem. J.* **253**, 287–289 (1988).
5. Seely, R. The energetics of electron transfer of chlorophyll and other compounds. *Photochem. Photobiol.* **27**, 639–654 (1978).
6. Barnese, K., Gralla, E. B., Valentine, J. S. & Cabelli, D. E. Biologically relevant mechanism for catalytic superoxide removal by simple manganese compounds. *Proc. Natl. Acad. Sci. U. S. A.* **109**, 6892–6897 (2012).
7. Scarpa, M., Stevanatos, R., Vigiinos, P. & Rig, A. Superoxide ion as active intermediate in the autoxidation of ascorbate by molecular oxygen. *J. Biol. Chem.* **258**, 6695–6697 (1983).
8. Nishikimi, M. Oxidation of ascorbic acid with superoxide anion generated by the xanthine-xanthine oxidase system. *Biochem. Biophys. Res. Commun.* **63**, 463–468 (1975).
9. Augusto, O. & Miyamoto, S. in *Principles of free radical biomedicine* (ed. Pantopoulos, K., Schipper, H.) (Nova Science, New York, 2011).
10. Cannella, D. *et al.* Light-driven oxidation of polysaccharides by photosynthetic pigments and a metalloenzyme. *Nat. Comm.* **7**, (2016).
11. Li, X. Improved pyrogallol autoxidation method: a reliable and cheap superoxide-scavenging assay suitable for all antioxidants. *J. Agric. Food Chem.* **60**, 6418–6424 (2012).
