# Supplementary material for: Controlled depolymerization of cellulose by light-driven lytic polysaccharide oxygenases
Source: Nat Commun. 2020 Feb 14;11:890. doi: 10.1038/s41467-020-14744-9 (PMC7021734; doi:10.1038/s41467-020-14744-9)
Supplement: Supplementary file 2 — Reporting Summary [file 41467_2020_14744_MOESM2_ESM.pdf]

nature research

Corresponding author(s): Vincent G.H. Eijsink

Last updated by author(s): Jan 20, 2020

Reporting Summary

Nature Research wishes to improve the reproducibility of the work that we publish. This form provides structure for consistency and transparency in reporting. For further information on Nature Research policies, see Authors & Referees and the Editorial Policy Checklist.

Statistics

For all statistical analyses, confirm that the following items are present in the figure legend, table legend, main text, or Methods section.

n/a	Confirmed	
☐	☒	The exact sample size (n) for each experimental group/condition, given as a discrete number and unit of measurement
☐	☒	A statement on whether measurements were taken from distinct samples or whether the same sample was measured repeatedly
☒	☐	The statistical test(s) used AND whether they are one- or two-sided<br>*Only common tests should be described solely by name; describe more complex techniques in the Methods section.*
☒	☐	A description of all covariates tested
☒	☐	A description of any assumptions or corrections, such as tests of normality and adjustment for multiple comparisons
☐	☒	A full description of the statistical parameters including central tendency (e.g. means) or other basic estimates (e.g. regression coefficient) AND variation (e.g. standard deviation) or associated estimates of uncertainty (e.g. confidence intervals)
☒	☐	For null hypothesis testing, the test statistic (e.g. F, t, r) with confidence intervals, effect sizes, degrees of freedom and P value noted<br>*Give P values as exact values whenever suitable.*
☒	☐	For Bayesian analysis, information on the choice of priors and Markov chain Monte Carlo settings
☒	☐	For hierarchical and complex designs, identification of the appropriate level for tests and full reporting of outcomes
☒	☐	Estimates of effect sizes (e.g. Cohen's d, Pearson's r), indicating how they were calculated

Our web collection on statistics for biologists contains articles on many of the points above.

Software and code

Policy information about availability of computer code

Data collection	not relevant for this study
Data analysis	not relevant for this study

For manuscripts utilizing custom algorithms or software that are central to the research but not yet described in published literature, software must be made available to editors/reviewers. We strongly encourage code deposition in a community repository (e.g. GitHub). See the Nature Research guidelines for submitting code & software for further information.

Data

Policy information about availability of data

All manuscripts must include a data availability statement. This statement should provide the following information, where applicable:

- Accession codes, unique identifiers, or web links for publicly available datasets
- A list of figures that have associated raw data
- A description of any restrictions on data availability

Data supporting the findings of this work are available within the paper and its Supplementary Information files. A reporting summary for this Article is available as a Supplementary Information file. The source data underlying Figures 2, 3, 4, 5, 6, 7, 8 as well as Supplementary Figures 3, 5, 7, 8, 9, 10, 11, and Supplementary Table 1 are available as a Source Data file in Figshare (10.6084/m9.figshare.11566926).

Field-specific reporting

Please select the one below that is the best fit for your research. If you are not sure, read the appropriate sections before making your selection.

☒ Life sciences ☐ Behavioural & social sciences ☐ Ecological, evolutionary & environmental sciences

For a reference copy of the document with all sections, see nature.com/documents/nr-reporting-summary-flat.pdf

Life sciences study design

All studies must disclose on these points even when the disclosure is negative.

Sample size	Sample sizes (= number of replicate experiments) were chosen according to common practices in enzyme research and were such that standard deviations were small enough to allow determination of significant effects and trends. In most cases, at least 3 independent replicates were carried out for each experiment. Only 1 replicate was performed when the experiment only aimed at screening reaction conditions.
Data exclusions	Some data points were excluded when they clearly appeared as outliers on the basis of the full set of data. This is visible in the Source Data file.
Replication	All experiments were done in duplicate or triplicate, as indicated in the manuscript (independent experiments). The data presented represent averages of independent experiments; all experimental data are included in these averages (no data exclusions). The screening experiments in Fig. S7, S8 & S9 are exceptions; these experiments were done only once and there are no standard deviations. Still, due to the testing of multiple timepoints and concentrations, the conclusions of these experiments are clearly significant.
Randomization	Not relevant for this study.
Blinding	Not relevant for this study.

Reporting for specific materials, systems and methods

We require information from authors about some types of materials, experimental systems and methods used in many studies. Here, indicate whether each material, system or method listed is relevant to your study. If you are not sure if a list item applies to your research, read the appropriate section before selecting a response.

Materials & experimental systems

n/a	Involved in the study
☒	☐ Antibodies
☒	☐ Eukaryotic cell lines
☒	☐ Palaeontology
☒	☐ Animals and other organisms
☒	☐ Human research participants
☒	☐ Clinical data

Methods

n/a	Involved in the study
☒	☐ ChIP-seq
☒	☐ Flow cytometry
☒	☐ MRI-based neuroimaging
